# Dynamic TyG trajectories cumulative TyG burden are associated with in-hospital mortality in acute brain injury: a multicenter interpretable machine-learning analysis

**DOI:** 10.3389/fnut.2026.1761240

**Published:** 2026-02-27

**Authors:** Juan Wang, Zheng Peng, Man-Man Xu, Meng-Lian Duan, Chun-Hua Hang, Peng-Lai Zhao

**Affiliations:** 1 Department of Neurosurgery, Nanjing Drum Tower Hospital Clinical College of Nanjing University of Chinese Medicine, Nanjing, China; 2 Department of Neurosurgery, Nanjing Drum Tower Hospital, Affiliated Hospital of Medical School, Nanjing University, Nanjing, China; 3 Neurosurgical Institute, Nanjing University, Nanjing, China

**Keywords:** Acute brain injury, interpretable machine learning, metabolic trajectory, threshold-based mean area under the curve, time-stratified Cox, triglyceride-glucose index

## Abstract

**Background:**

Dynamic metabolic changes may influence outcomes after acute brain injury (ABI), but most ICU studies use only a single triglyceride–glucose (TyG) value. We examined whether ICU TyG trajectories and a cumulative TyG burden provide time-sensitive prognostic information and can be embedded in an interpretable mortality model.

**Methods:**

Adults with ABI from three ICU databases (NSICU, MIMIC-IV, eICU) were retrospectively analyzed. TyG trajectories were derived from serial ICU measurements, cumulative exposure was summarized as prespecified threshold-based mean area under the curve (TBM), and in-hospital mortality was evaluated with 7-day time-stratified Cox models. A machine-learning model including TyG trajectory, TBM, and routinely available clinical variables was trained in NSICU and validated in the pooled external cohort.

**Results:**

Among 4,760 admissions, three trajectories were identified—low–slightly increasing (LSI), moderate–increasing (MI), and persistently high (PH). Mortality did not differ across trajectories during days 0–7, but after day 7 both MI (HR 1.48, 95% CI 1.18–1.86; *P* < 0.001) and PH (HR 1.51, 95% CI 1.17–1.93; *P* = 0.001) showed higher in-hospital mortality than LSI. TBM showed a parallel positive association; TBM8p7 remained significant in fully adjusted models (HR 1.42, 95% CI 1.18–1.70; *P* < 0.001). ExtraTrees was selected for its consistent internal and external validation performance, and model interpretability analyses placed TyG trajectory and TBM8p7 among the next most important predictors alongside SOFA score and vasopressor use.

**Conclusion:**

In ICU-treated ABI, TyG is better modeled as a time-aware exposure: trajectory differences become prognostically relevant only after the first week, whereas cumulative TBM8p7 shows a graded, independent association with mortality. Both metrics add risk information beyond conventional severity indicators and can be integrated into an interpretable, externally tested model.

## Graphical Abstract

**Figure:**
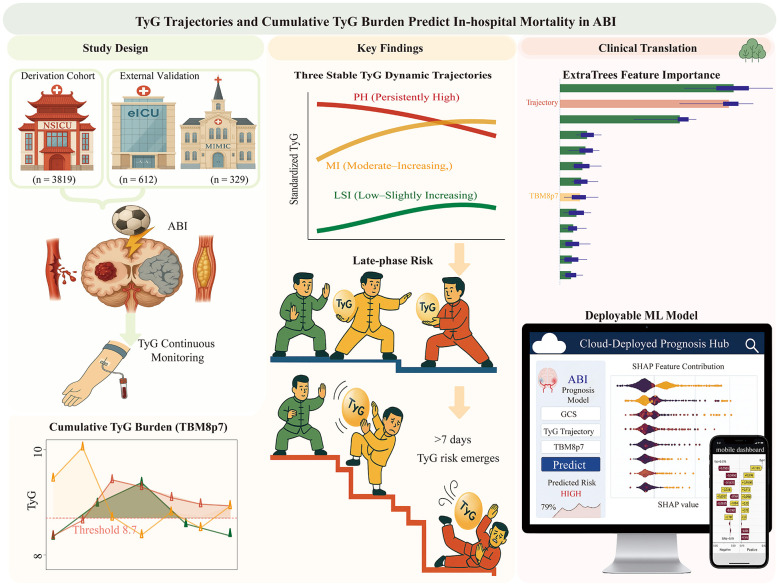
ICU ABI patients from a derivation cohort (NSICU, n = 3,819) and a pooled external cohort (MIMIC-IV + eICU, n = 941) had early serial TyG measured. LCGM identified three reproducible TyG trajectories (LSI, MI, PH); in a 7-day landmark analysis, mortality curves began to diverge after day 7. A continuous burden metric, TBM8p7 (threshold-based mean supra-threshold area above 8.7 across observed intervals), was also derived. These dynamic metabolic features were incorporated into the prediction model, which was deployed as a web/mobile tool with SHAP-based individual explanations.

## 1 Introduction

Acute brain injury (ABI), mainly acute stroke and traumatic brain injury (TBI), remains among the most lethal and disabling entities in neurocritical care. Recent Global Burden of Disease reports indicate that stroke ranks third for mortality and fourth for disability worldwide, while TBI continues to affect millions and leaves many survivors with long-term sequelae (1, 2). Beyond the focal cerebral insult, ABI provokes a centrally driven stress response with neuroendocrine and metabolic activation that has been linked to systemic immune–metabolic dysregulation (3, 4). Evidence from major ICU glucose-control trials and the 2024 SCCM guideline shows that stress-related dysglycemia is clinically relevant but should not be suppressed indiscriminately, highlighting the absence of bedside approaches that distinguish transient stress hypermetabolism from sustained dysmetabolism (5–7).

In parallel, the triglyceride–glucose (TyG) index has become a widely used and low-cost surrogate of insulin resistance. Large population-based and multicontinent cohorts have shown that higher TyG predicts incident stroke and can partly mediate the effect of excess adiposity or metabolic load on cerebrovascular risk (8–10). A recent meta-analysis reported consistent associations between TyG and a broad range of cardiometabolic and cerebrovascular outcomes, supporting TyG as a risk-bearing metabolic marker (11). In the ICU setting, database studies of critically ill stroke patients have likewise found that higher admission TyG is associated with ICU or in-hospital mortality across independent datasets, including MIMIC-IV and eICU (12–14).

However, analyses from other populations, including work that incorporated Mendelian randomization, have yielded directionally opposite estimates, suggesting that a single TyG measurement is sensitive to timing and case mix and may not distinguish ABI-related, short-lived surges from sustained metabolic activation (15). By contrast, longitudinal studies of TyG trajectories and cohorts evaluating cumulative TyG exposure have shown that patterns that remain high or rise over time are related to later cardiovascular or cerebrovascular events in a graded, dose–response fashion (16–18). Taken together, these findings suggest that, in ABI, TyG needs to be characterized over time to capture the clinically relevant metabolic burden.

Accordingly, we aimed to delineate reproducible TyG trajectories across three ICU databases, assess whether trajectories and cumulative TyG burden relate to in-hospital mortality in a time-dependent manner, and evaluate whether adding these dynamic metrics improves explainable machine learning (ML) models in ICU-treated ABI due to acute stroke (ischemic or hemorrhagic) or TBI.

## 2 Methods

### 2.1 Study design and cohorts

**Figure 1:**
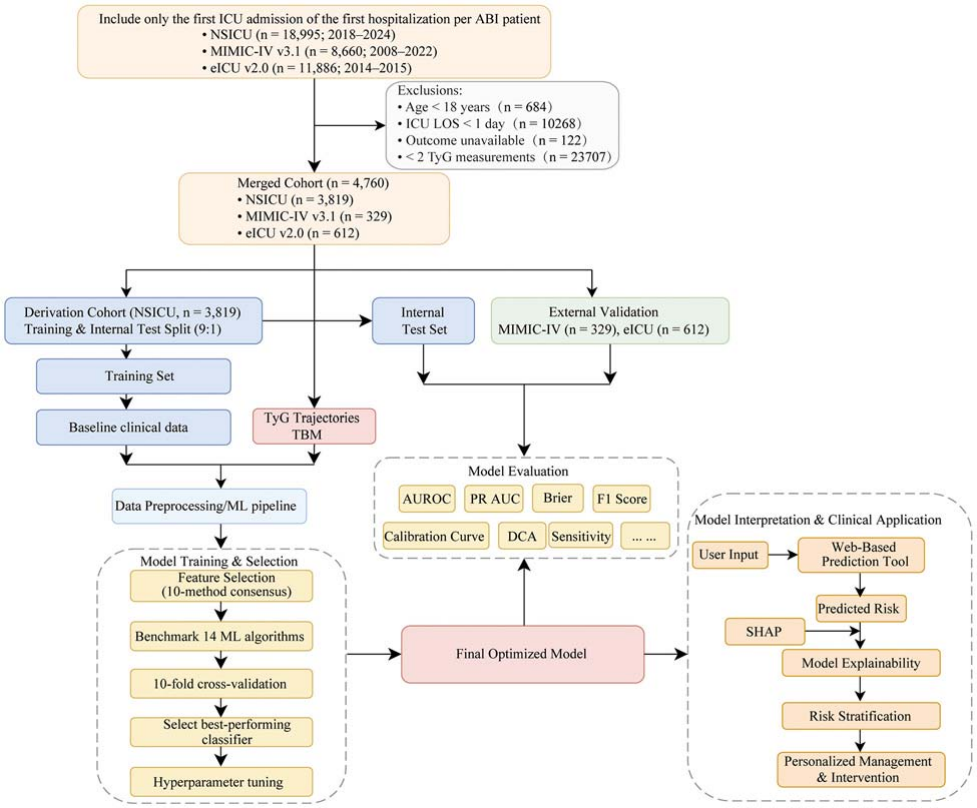
Flowchart of participant selection and model development. Patients from three ICU databases (NSICU, MIMIC-IV, and eICU) were screened using prespecified criteria to derive a merged cohort. The NSICU cohort was split (9:1) into training and internal test sets, and MIMIC-IV plus eICU served as external validation. TyG trajectories and TBM were integrated with baseline clinical variables to develop and evaluate the final model.

We conducted a multicenter, retrospective cohort study using NSICU, MIMIC-IV v3.1, and eICU-CRD v2.0. Adults (≥18 years) with a primary ICU diagnosis of ABI (ischemic stroke, hemorrhagic stroke, or TBI) were included; only the first ICU admission per hospitalization was analyzed. We excluded admissions with ICU length of stay < 24 h, missing in-hospital outcome, or fewer than two TyG measurements during the ICU stay (Figure 1). NSICU was used for model derivation with a 9:1 random split into training and internal test sets, and the pooled MIMIC-IV/eICU cohort was used exclusively for external validation. Use of de-identified data was approved for all sources; details are provided in the Ethics section. Reporting adhered to STROBE and TRIPOD recommendations for prognostic model development with external validation.

### 2.2 Data elements and definitions

We extracted a harmonized set of ICU variables from NSICU, MIMIC-IV, and eICU to allow cross-cohort analyses (Supplementary Table 1). Baseline missingness is summarized in Supplementary Table 2, and imputation diagnostics are shown in Supplementary Figures 1–2. Variables included demographics and admission characteristics (age, sex, BMI, trauma status, ABI subtype); comorbidities [hypertension (HTN), diabetes mellitus (DM), chronic kidney disease (CKD), liver disease (LD), CCI]; acute severity scores (SOFA, APACHE III, GCS at ICU admission); physiological measurements around ICU admission [temperature, respiratory rate (RR), heart rate (HR), mean blood pressure (MBP)]; and initial ICU interventions [mechanical ventilation (MV), vasopressor use, mannitol or sedation, renal replacement therapy (RRT), and neurosurgical or endovascular procedures]. ICU laboratory data included complete blood count, coagulation tests, basic chemistries, glucose, and triglycerides. The TyG index was calculated as TyG = ln [triglycerides (mg/dL) × glucose (mg/dL)/2] after harmonizing triglyceride and glucose units to mg/dL.

### 2.3 Dynamic TyG characterization

#### 2.3.1 Trajectory modeling of serial TyG

For each patient, serial TyG measurements during the ICU stay were ordered chronologically, and up to the first seven measurements were retained; patients with fewer than two measurements had already been excluded during cohort assembly. To account for between-database laboratory scale differences, TyG values were standardized to *z*-scores within each database (mean 0, SD 1) before modeling. Latent class growth modeling (LCGM) was then applied to the pooled standardized series, evaluating candidate solutions with 2–6 classes. Model selection was guided by AIC, BIC, sample-size–adjusted BIC, entropy, minimum class size, and clinical interpretability, and each patient was assigned to the class with the highest posterior probability. To assess robustness of the class structure, the same procedure was repeated separately in NSICU, MIMIC-IV, and eICU. The accumulation and timing of TyG measurements are shown in Supplementary Figure 3.

#### 2.3.2 Threshold-based TyG burden (TBM)

Using the same ordered TyG series (2–7 measurements per patient), we constructed a threshold-based mean area under the curve (TBM) to quantify cumulative TyG exposure (Supplementary Figure 4). Prespecified TyG thresholds (8.0, 8.3, 8.5, 8.7, 9.0, 9.5) were selected *a priori* based on published cut points (12, 13, 19, 20) and were checked against the empirical TyG distribution in our cohort to ensure adequate coverage and feasibility of supra-threshold exposure across thresholds. For each threshold, only the excess of TyG above the threshold was integrated over adjacent measurement intervals using a zero-truncated trapezoidal rule, and the resulting area was divided by the number of observed intervals (N−1, where *N* is the number of TyG measurements for that patient) to obtain a patient-level TBM at that threshold.

### 2.4 Statistical analyses

Baseline characteristics were summarized as mean (SD) or median (IQR) for continuous variables and as n(%) for categorical variables. Groups defined by TyG trajectory were compared using ANOVA or Kruskal–Wallis tests for continuous variables and χ^2^ or Fisher's exact tests for categorical variables, as appropriate. Missing baseline covariates were imputed by multiple imputation using chained equations (*m* = 10) under a missing-at-random assumption, and estimates were pooled using Rubin's rules.

Survival time was measured from ICU admission to in-hospital death, with patients censored at hospital discharge. Survival was described using Kaplan–Meier curves and compared with the log-rank test. Cox proportional hazards models were fitted with TyG trajectory and TBM (per 1-unit increase in the corresponding TBM) as the main exposures. The proportional hazards assumption was evaluated using Schoenfeld residuals; when a violation was detected, effects were re-estimated in a prespecified two-interval Cox model (0–7 days and >7 days), and hazard ratios (HRs) with 95% confidence intervals (CIs) were reported for each interval.

Covariates were selected *a priori* based on ABI literature and clinical relevance. Variables were retained if they were considered clinically essential or if they changed the exposure estimate by ≥10%. Associations were reported in three stages: Model 1 unadjusted; Model 2 adjusted for demographics and comorbidities; and Model 3 additionally adjusted for admission severity and initial ICU treatments. The same modeling strategy was applied to all prespecified TBM thresholds. Subgroup and interaction analyses were prespecified for age (< 65 vs. ≥65 years), trauma status (traumatic vs. non-traumatic ABI), HTN, craniotomy, and data source (NSICU vs. pooled MIMIC-IV/eICU). All analyses were performed in R (version 4.2.2) and the Free Statistics analysis platform (21). Two-sided *P* values < 0.05 were considered statistically significant.

### 2.5 Feature selection, model development, and interpretability

We first defined a candidate predictor set consisting of demographics and admission characteristics, comorbidities, acute severity scores, key ICU interventions, routine laboratory variables, and the two prespecified metabolic exposures (TyG trajectory class and TBM). This full set was screened using 10 complementary feature-selection procedures [least absolute shrinkage and selection operator (LASSO), Boruta, recursive feature elimination (RFE), and seven filter/wrapper methods based on information gain, mutual information, permutation, or model performance]. Predictors that were selected by at least five of these procedures and were available in all three databases were retained as the final ICU-feasible feature set.

Using this fixed feature set, we benchmarked 14 supervised classifiers—logistic regression (Logistic), classification and regression tree (CART), k-nearest neighbors (k-NN), Naive Bayes classifier (Naive Bayes), support vector machine (SVM), multilayer perceptron neural network (Neural Network), gradient boosting machine (GBM), Adaptive Boosting (AdaBoost), Extreme Gradient Boosting (XGBoost), Light Gradient Boosting Machine (LightGBM), Categorical Boosting (CatBoost), random forest (Random Forest), Extremely Randomized Trees (ExtraTrees), and a Bayesian network (Bayes Net)—by stratified 10-fold cross-validation in the NSICU training set. Within each fold, imputation, scaling or encoding, model fitting, and hyperparameter tuning were performed inside the resampling loop to avoid information leakage. Tuned models were then evaluated on the held-out NSICU internal test set, and the classifier showing the most stable performance with the most favorable and stable overall profile in cross-validation and the internal test set. This locked model was subsequently applied, without refitting, to the pooled external validation cohort (MIMIC-IV plus eICU).

Model interpretability was assessed using SHAP to obtain global and patient-level feature attributions, and using model-agnostic permutation- and loss-based importance (DALEX/IML) to quantify the performance decrement after shuffling individual predictors.

## 3 Results

A total of 4,760 ICU admissions for ABI from NSICU, MIMIC-IV, and eICU were included (Figure 1). NSICU (*n* = 3,819) served as the derivation cohort and was split 9:1 into a training set (*n* = 3,437) and an internal test set (*n* = 382). MIMIC-IV (*n* = 329) and eICU (*n* = 612) were combined as the pooled external validation cohort (*n* = 941).

### 3.1 TyG Trajectories and Cohort Characteristics

**Figure 2:**
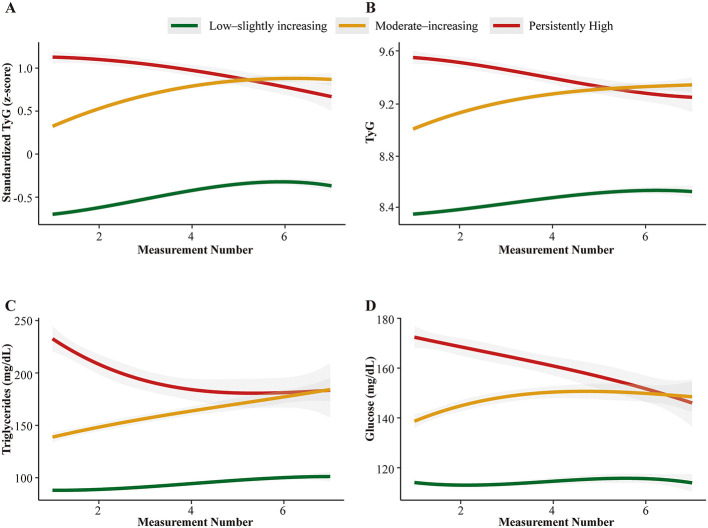
TyG trajectory phenotypes in the integrated cohort. **(A–D)** display the standardized TyG z-score, raw TyG, triglycerides, and glucose across measurement numbers 1–7. Solid lines indicate model-estimated mean trajectories, and shaded ribbons show 95% confidence bands. Trajectory 1 is Low–slightly increasing (LSI, green), Trajectory 2 is Moderate–increasing (MI, yellow), and Trajectory 3 is Persistently High (PH, red).

LCGM applied to serial ICU TyG measurements in the integrated cohort identified three distinct and clinically interpretable classes (Figure 2). A three-class solution provided the best compromise between model fit (log-likelihood, AIC, BIC, SABIC), entropy, and acceptable class sizes. Models with ≥4 classes sometimes improved information criteria but generated very small or poorly separated groups and were therefore not retained. The three classes accounted for 61.8%, 23.5%, and 14.8% of patients, respectively, with reasonable posterior assignment (Supplementary Table 3). Running LCGM separately in NSICU, MIMIC-IV, and eICU yielded the same three-class structure, supporting cross-cohort stability (Supplementary Tables 4–6).

The classes were labeled according to temporal patterns: trajectory 1, low–slightly increasing (LSI); trajectory 2, moderate–increasing (MI); and trajectory 3, persistently high (PH). LSI showed low TyG with only a mild rise; MI started at an intermediate level and increased more clearly; PH began at the highest level and declined only slightly, approaching and partly overlapping with MI at later measurements, while both stayed clearly above LSI (Figure 2; Supplementary Figures 5–7), and a sensitivity analysis using 10 sequential measurements showed consistent class patterns with wider uncertainty at later measurements (Supplementary Figure 8). Because TyG came from three databases with different laboratory distributions, LCGM was performed on standardized values; class membership was then displayed on raw TyG and on its components (triglycerides and glucose), and the three patterns remained visually distinct.

Baseline characteristics showed a parallel clinical gradient across trajectories (Table 1; Supplementary Table 7). LSI had the most favorable profile, with lower BMI and the lowest prevalence of diabetes (8.6%) and hypertension (56.8%). PH concentrated adverse features, including higher BMI, more diabetes (39.1%) and hypertension (71.4%), and higher acute severity scores, while MI showed intermediate values. A similar gradient was seen in ICU interventions: MV was used in 41.5% of LSI and 66.1% of PH. These patterns indicate that TyG-based latent classes map onto clinically meaningful ICU phenotypes rather than modeling artifacts.

**Table 1:** Baseline characteristics by TyG trajectory.

**Characteristics**	**Total**	**Low–slightly increasing**	**Moderate–increasing**	**Persistently high**	***P***
	**(*****n*** = **4,760)**	**(*****n*** = **2,940)**	**(*****n*** = **1,117)**	**(*****n*** = **703)**	
**Demographics & admission**					
Age (years)	57.6 ± 14.5	58.0 ± 15.2	57.6 ± 13.4	56.1 ± 13.2	0.010
Sex, Male	2,796 (58.7)	1,708 (58.1)	656 (58.7)	432 (61.5)	0.268
BMI (kg/m^2^)	24.7 (22.2, 27.7)	24.0 (21.5, 26.7)	25.7 (23.1, 28.7)	26.1 (23.8, 29.4)	<0.001
Trauma	1,053 (22.1)	655 (22.3)	230 (20.6)	168 (23.9)	0.241
HR (bpm)	84.2 ± 20.1	81.8 ± 19.1	85.9 ± 19.9	91.1 ± 22.3	<0.001
**Physiological & laboratory**					
BUN (mg/dL)	13.9 (10.6, 18.5)	13.2 (10.2, 17.0)	14.6 (11.2, 19.6)	16.0 (12.0, 22.7)	<0.001
Cr (mg/dL)	0.7 (0.6, 0.9)	0.7 (0.6, 0.8)	0.7 (0.6, 0.9)	0.8 (0.6, 1.1)	<0.001
Sodium (mmol/L)	140.7 ± 5.1	140.4 ± 4.6	141.1 ± 5.7	141.3 ± 6.1	<0.001
**Comorbidities**					
HTN	2,947 (61.9)	1,671 (56.8)	774 (69.3)	502 (71.4)	<0.001
DM	831 (17.5)	253 (8.6)	303 (27.1)	275 (39.1)	<0.001
CKD	853 (17.9)	425 (14.5)	249 (22.3)	179 (25.5)	<0.001
CCI	3.0 (2.0, 5.0)	3.0 (2.0, 5.0)	4.0 (2.0, 6.0)	4.0 (2.0, 6.0)	<0.001
**Severity & ICU interventions**					
SOFA	4.0 (2.0, 6.0)	4.0 (1.0, 5.0)	4.0 (2.0, 6.0)	5.0 (3.0, 6.0)	<0.001
APACHE III	65.9 ± 19.8	63.5 ± 19.8	68.6 ± 18.7	71.9 ± 19.9	<0.001
GCS	9.0 ± 5.2	9.8 ± 5.2	7.9 ± 5.0	7.4 ± 4.8	<0.001
MV	2,353 (49.4)	1,220 (41.5)	668 (59.8)	465 (66.1)	<0.001
Embolization	1,836 (38.6)	1,263 (43.0)	379 (33.9)	194 (27.6)	<0.001
Vaso	532 (11.2)	293 (10.0)	145 (13.0)	94 (13.4)	0.003
Craniotomy	1,948 (40.9)	1,129 (38.4)	502 (44.9)	317 (45.1)	<0.001
**Outcomes**					
Hospital LOS (days)	14.0 (9.7, 19.6)	13.0 (9.0, 18.8)	14.8 (10.8, 21.0)	16.0 (11.0, 21.5)	<0.001
ICU LOS (days)	10.5 (4.6, 16.5)	8.6 (3.6, 15.1)	12.7 (7.3, 18.3)	12.8 (7.8, 18.8)	<0.001
In-hospital mortality	597 (12.5)	275 (9.4)	182 (16.3)	140 (19.9)	<0.001

Continuous variables are summarized as mean ± SD or median (IQR); categorical variables as *n* (%). For binary variables, *n* (%) indicates the presence of the characteristic (Yes); for sex, values indicate males. TyG, triglyceride–glucose index; BMI, body mass index; HR, heart rate; BUN, blood urea nitrogen; Cr, creatinine; HTN, hypertension; DM, diabetes mellitus; CKD, chronic kidney disease; CCI, Charlson Comorbidity Index; SOFA, Sequential Organ Failure Assessment; APACHE III, Acute Physiology and Chronic Health Evaluation III; GCS, Glasgow Coma Scale; MV, mechanical ventilation; Vaso, vasopressor use; LOS, length of stay.

### 3.2 Time-stratified prognostic associations of TyG trajectories and TBM

**Figure 3:**
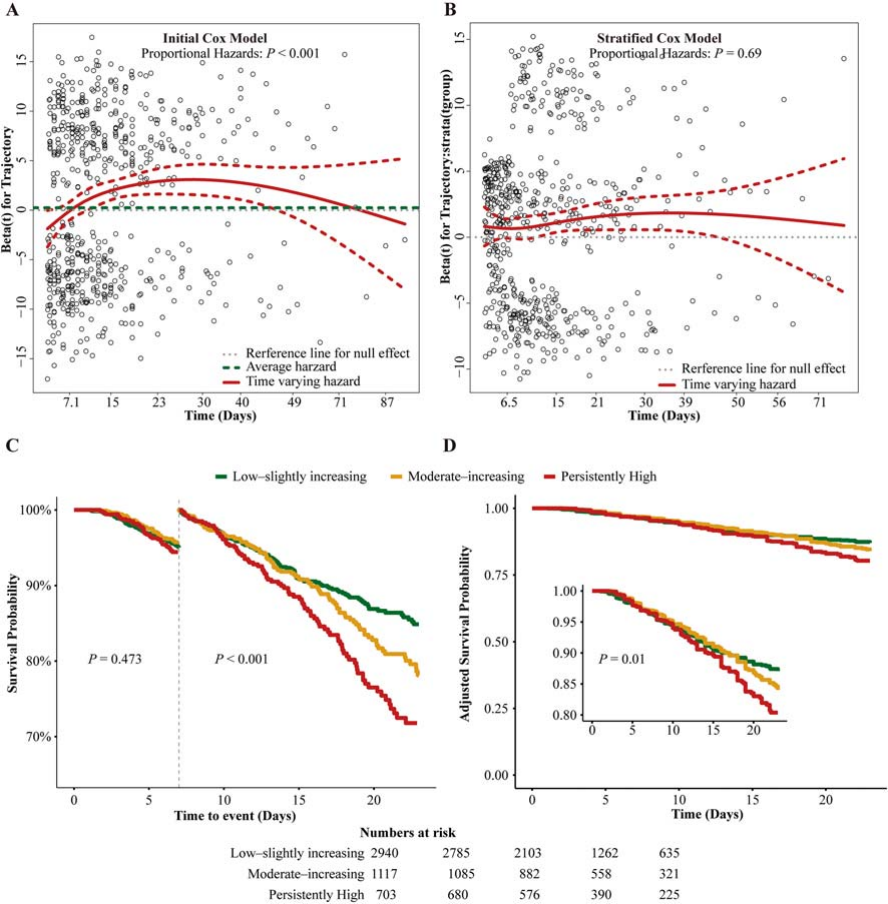
Proportional hazards diagnostics and landmark survival by Tyg trajectory. **(A, B)** Schoenfeld residual diagnostics for TyG trajectories: the unstratified Cox model violates proportional hazards (*P* < 0.001), whereas the 7-day time-stratified Cox model (0–7 vs. >7 days) does not (*P* = 0.69). The >7-day comparison is conditional on the 7-day landmark. **(C)** Landmark Kaplan–Meier curves with log-rank *P*-values for 0–7 days and >7 days. **(D)** Covariate-adjusted survival from the fully adjusted stratified Cox model (inset: 0–7 days). Colors: LSI (green), MI (yellow), PH (red).

The proportional-hazards assumption was not met for TyG trajectories in the unstratified Cox model (Schoenfeld *P* < 0.001; Figure 3A), so we refitted a 7-day time-stratified Cox model, for which the test of proportional hazards was acceptable (*P* = 0.69; Figure 3B). This specification matched the landmark Kaplan–Meier curves: trajectories showed minimal separation during days 0–7 and then diverged in an ordered fashion after day 7, with PH having the lowest survival, LSI the highest, and MI intermediate; covariate-adjusted survival curves showed the same pattern (Figures 3C–D).

The candidate covariate set for multivariable adjustment was defined *a priori* and was supported by change-in-estimate screening with collinearity diagnostics (Supplementary Table 8). Time-stratified Cox estimates quantified this pattern (Table 2). During days 0–7, neither MI (adjusted HR 0.85, 95% CI 0.58–1.24) nor PH (adjusted HR 0.99, 95% CI 0.65–1.50) differed from LSI. After day 7, both higher trajectories were associated with increased in-hospital mortality: MI, HR 1.48 (95% CI 1.18–1.86); PH, HR 1.51 (95% CI 1.17–1.93). TBM showed a parallel dose–response: all prespecified TBM thresholds were positively associated with mortality, and effect sizes rose with higher thresholds. In the fully adjusted model, TBM8p7 (a prespecified threshold) gave a consistent estimate (HR 1.42, 95% CI 1.18–1.70). Sensitivity analyses were consistent, whether using 10-measurement trajectories or alternative stratification schemes, with risk concentrated after the early interval (Supplementary Tables 9–10).

**Table 2:** Time-stratified cox models for in-hospital mortality by Tyg trajectory and TBM.

**Variable**	**Model 1**		**Model 2**		**Model 3**	
	**HR (95% CI)**	***P***	**HR (95% CI)**	***P***	**HR (95% CI)**	***P***
**Time interval: 0–7 days**					
Low–slightly increasing	Reference		Reference		Reference	
Moderate–increasing	1.11 (0.77–1.61)	0.585	1.02 (0.70–1.48)	0.928	0.85 (0.58–1.24)	0.397
Persistently high	1.37 (0.91–2.06)	0.129	1.23 (0.81–1.87)	0.322	0.99 (0.65–1.50)	0.971
**Time interval:** >**7 days**					
Low–slightly increasing	Reference		Reference		Reference	
Moderate–increasing	1.70 (1.37–2.12)	<0.001	1.58 (1.26–1.97)	<0.001	1.48 (1.18–1.86)	<0.001
Persistently high	1.84 (1.45–2.33)	<0.001	1.75 (1.36–2.24)	<0.001	1.51 (1.17–1.93)	0.001
**TBM thresholds (per 1-unit increase)**					
TBM8p0	1.61 (1.42–1.82)	<0.001	1.51 (1.31–1.73)	<0.001	1.30 (1.12–1.51)	0.001
TBM8p3	1.66 (1.46–1.90)	<0.001	1.56 (1.34–1.81)	<0.001	1.34 (1.14–1.57)	<0.001
TBM8p5	1.72 (1.49–1.98)	<0.001	1.61 (1.38–1.88)	<0.001	1.38 (1.16–1.63)	<0.001
TBM8p7	1.79 (1.54–2.09)	<0.001	1.67 (1.41–1.98)	<0.001	1.42 (1.18–1.70)	<0.001
TBM9p0	1.94 (1.62–2.33)	<0.001	1.79 (1.47–2.19)	<0.001	1.50 (1.21–1.86)	<0.001
TBM9p5	2.43 (1.85–3.20)	<0.001	2.20 (1.66–2.93)	<0.001	1.77 (1.29–2.43)	<0.001

TyG trajectory used Low–slightly increasing (LSI) as the reference. *P*-values are from Wald tests for the coefficient shown (trajectory contrasts vs. LSI, or TBM per 1-unit increase) within each model and time interval. Each TBM threshold was modeled separately using the same covariate set. Model 1 unadjusted; Model 2 adjusted for age, trauma, HR, BUN, Cr, sodium, CKD, HTN, DM, and CCI; Model 3 additionally adjusted for SOFA, APACHE III, GCS, MV, embolization, Vaso, and craniotomy.

**Figure 4:**
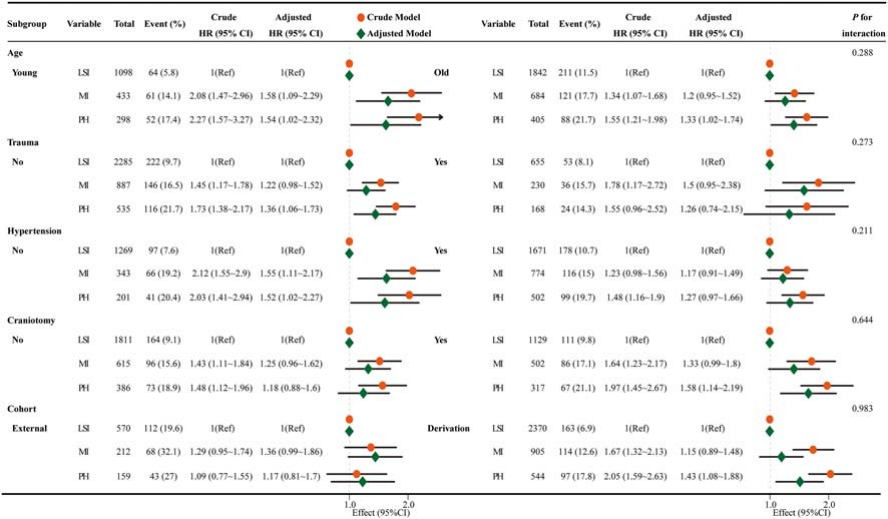
Subgroup hazard ratios by Tyg trajectory. Forest plots show hazard ratios (HRs) with 95% CIs for Moderate–increasing (MI) and Persistently High (PH) TyG trajectories compared with Low–slightly increasing (LSI) as the reference across prespecified subgroups. Orange circles denote crude HRs; green diamonds denote fully adjusted HRs. Horizontal bars indicate 95% CIs; the vertical dashed line marks HR = 1.

Subgroup analyses showed that the excess risk of MI and PH over LSI was preserved across age, trauma status, hypertension, craniotomy, and data-source strata (all interaction *P* > 0.10; Figure 4). The association between TBM8p7 and mortality was likewise positive in all subgroups; the only nominal interaction (derivation vs external, *P* = 0.01) was small and did not alter the direction of effect (Supplementary Figure 9).

### 3.3 Variable selection for machine-learning models

**Figure 5:**
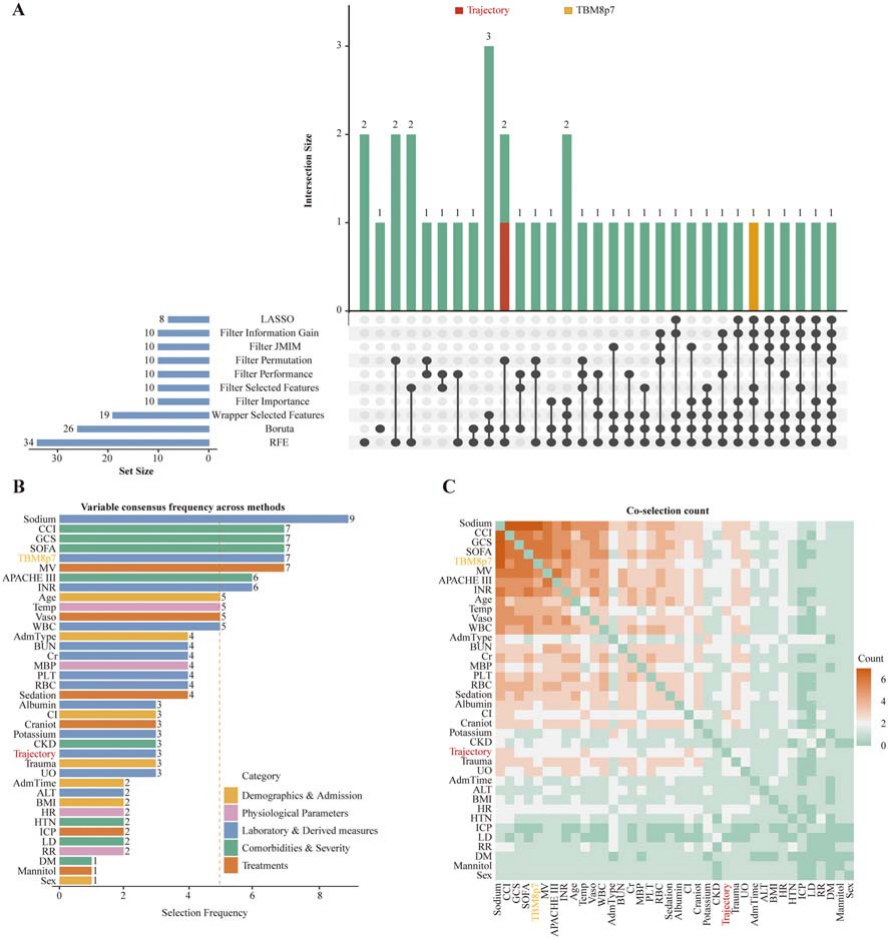
Feature selection consensus and co-selection across methods. **(A)** summarizes feature sets selected by multiple methods (LASSO, filter- and wrapper-based approaches, Boruta, and RFE), with set sizes and intersection sizes. **(B)** shows across-method selection frequency (colored by clinical domain); the dashed line indicates the prespecified consensus threshold (>5). **(C)** displays co-selection counts (darker = more frequent). Features carried forward to machine learning were those meeting the consensus threshold plus the prespecified dynamic features (TyG trajectory and TBM8p7): Age, Temperature, WBC, Sodium, INR, APACHE III, CCI, GCS, SOFA, Vaso, MV, TyG trajectory, and TBM8p7.

We applied 10 feature-selection procedures (LASSO, Boruta, recursive feature elimination, and seven additional filter/wrapper methods). Because individual procedures yielded partly discordant variable lists (Figure 5A), we defined a consensus as variables selected by at least 5 of the 10 methods (Figure 5B). This approach produced a compact, clinically interpretable panel consisting of sodium, age, temperature, WBC, INR, CCI, APACHE III, SOFA, GCS, mechanical ventilation, and vasopressor use. The two prespecified metabolic exposures, TyG trajectory and TBM8p7, were also retained. TBM8p7 met the consensus threshold, and TyG trajectory showed consistent co-selection with ICU severity variables in the heatmap (Figure 5C). All 13 variables were available in both the NSICU derivation cohort and the pooled external cohort (which showed slightly higher acuity; Supplementary Table 11), so this common feature set was used for subsequent benchmarking and validation. Adding TyG trajectory and TBM8p7 provided incremental predictive gain beyond baseline variables (Supplementary Figure 10).

### 3.4 Model development and benchmarking

**Figure 6:**
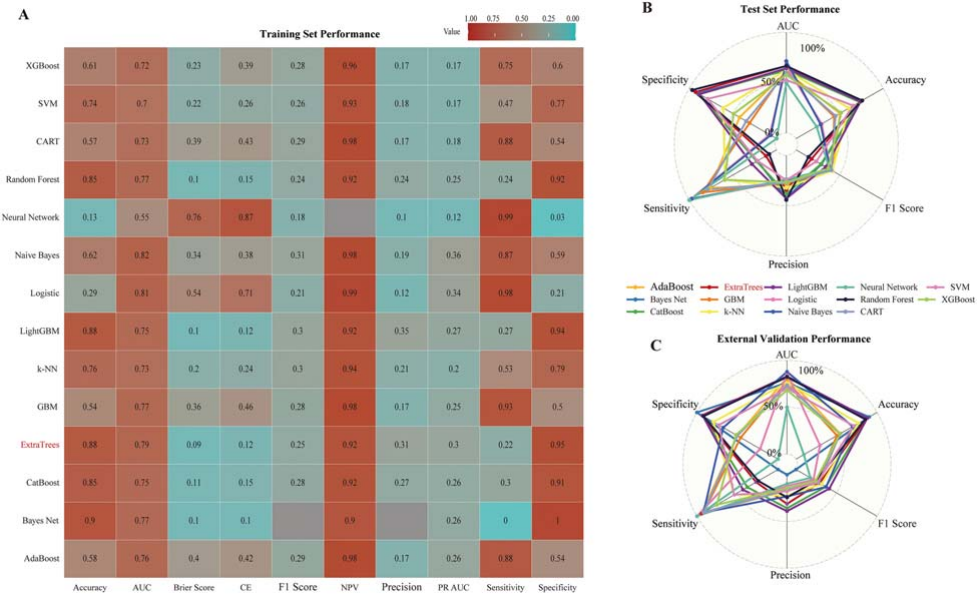
Classifier benchmarking and selection of Extratrees. **(A)** shows stratified 10-fold cross-validated performance on the training set for candidate classifiers across Accuracy, AUC, Brier score, classification error (CE), F1 score, NPV, Precision, PR AUC, Sensitivity, and Specificity; values are scaled to 0–1 for comparability, and lower is better for CE and Brier. **(B)** presents test set performance using a radar chart across the same metrics. **(C)** presents external-validation performance. Considering discrimination, error, precision–recall balance, and stability from test to external validation, Extratrees provided the most balanced profile and was selected for subsequent modeling and interpretation.

Fourteen classifiers were benchmarked using stratified 10-fold cross-validation in the NSICU training set (Figure 6A, Supplementary Figure 11, Supplementary Table 12). Tree-based ensemble classifiers (ExtraTrees, random forest) achieved higher discrimination and more stable performance across metrics than linear, probabilistic, or margin-based learners. When the same classifiers were evaluated on the held-out NSICU internal test set and on the pooled external validation cohort, ExtraTrees showed the smallest loss of performance, suggesting superior portability across datasets (Figures 6B–C, Supplementary Tables 13–14).

**Figure 7:**
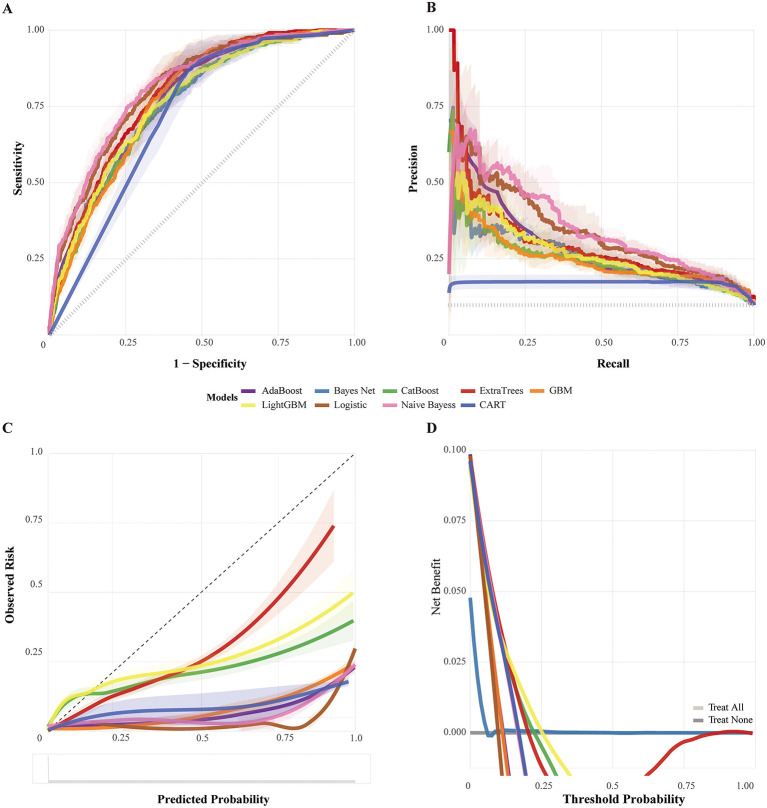
Cross-validated performance of benchmark classifiers in the training set. Results are from stratified 10-fold cross-validation within the training set; solid lines are fold means and shaded ribbons are 95% CIs. **(A)** ROC (sensitivity vs. 1–specificity). **(B)** Precision–recall (horizontal dashed line = outcome prevalence). **(C)** Calibration (LOESS-smoothed observed vs. predicted; diagonal = ideal). **(D)** Decision-curve analysis (net benefit vs. threshold probability; gray lines = treat-all and treat-none). Across CV, ExtraTrees showed the most favorable overall trade-off and was selected for downstream tuning.

**Figure 8:**
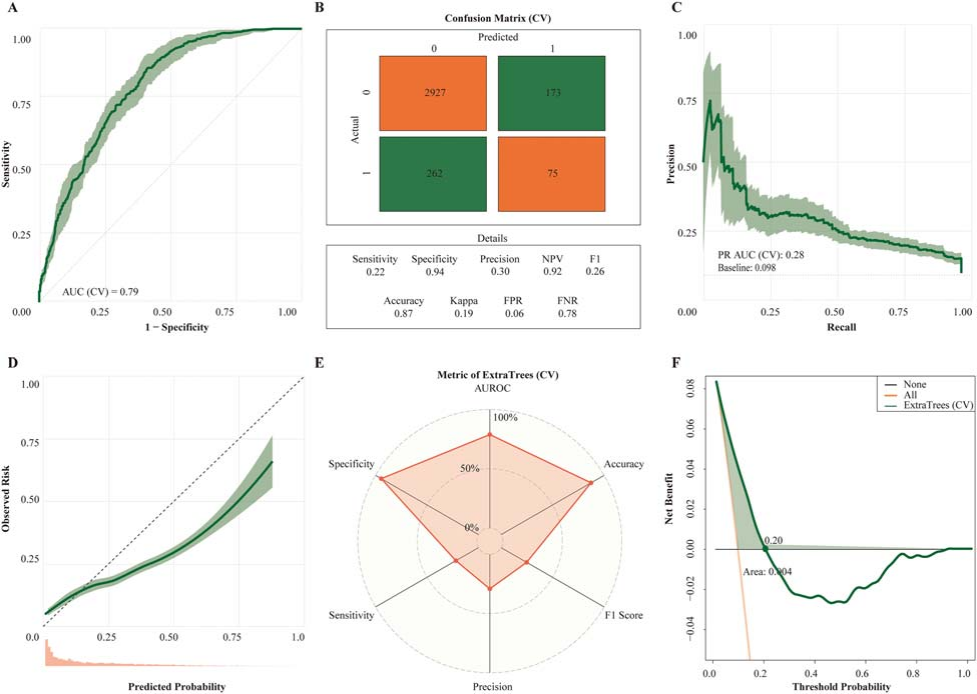
Cross-validated performance of the extratrees classifier in the training set. All panels summarize the ExtraTrees model under stratified 10-fold cross-validation in the derivation cohort; solid lines denote fold means and shaded ribbons the 95% CI. **(A)**, ROC curve with cross-validated AUC. **(B)**, Confusion matrix with Accuracy, Sensitivity (Recall), Specificity, Precision (PPV), F1 score, and Cohen's κ (chance-corrected agreement). **(C)**, Precision–recall curve with PR AUC; the horizontal dashed line marks the outcome prevalence (Prevalence). **(D)**, Calibration (LOESS-smoothed observed vs. predicted risk) with 45 ° reference; histogram of predicted probabilities shown below. **(E)**, Radar plot of cross-validated metrics (AUC, Accuracy, Sensitivity, Specificity, Precision, F1). **(F)**, Decision-curve analysis: net benefit vs. Threshold probability, with “Treat none” and “Treat all” reference strategies.

ExtraTrees was therefore selected as the final model. After hyperparameter tuning (Supplementary Table 15), the AUROC was 0.79 in cross-validation, 0.83 in the internal test set, and 0.66 in external validation; the corresponding accuracies were 0.87, 0.87, and 0.74, and the specificities 0.94, 0.94, and 0.93, respectively (Table 3). Benchmarking curves across classifiers are shown in Figure 7, whereas detailed performance of the selected ExtraTrees model is shown in Figure 8 (cross-validation) and Supplementary Figures 12–13 (internal test and external validation). Sensitivity analyses using a Youden index–optimized operating threshold are reported in Supplementary Table 16.

**Table 3:** Cross-validation, apparent training, and hold-out performance of the tuned extratrees classifier.

**Cohort**	**AUROC**	**Accuracy**	**Brier score**	**F1 score**	**CE**	**PR AUC**	**Sensitivity**	**Specificity**	**NPV**	**PPV**
CV	0.79	0.87	0.09	0.26	0.13	0.28	0.22	0.94	0.92	0.30
Training	1.00	0.98	0.01	0.92	0.02	0.98	0.91	0.99	0.99	0.94
Internal test	0.83	0.87	0.09	0.27	0.13	0.35	0.24	0.94	0.92	0.30
External validation	0.66	0.74	0.18	0.21	0.26	0.34	0.15	0.93	0.78	0.39

Metrics are for the final ExtraTrees classifier after hyperparameter tuning on the NSICU training set. “CV” values are out-of-fold means from stratified 10-fold cross-validation and reflect expected performance on unseen data. The “Training” row reports apparent (in-sample) performance of the fitted model. Internal test and external validation metrics were obtained out-of-sample using the same fitted model and a prespecified operating threshold from cross-validation.

### 3.5 Model interpretation and web deployment

**Figure 9:**
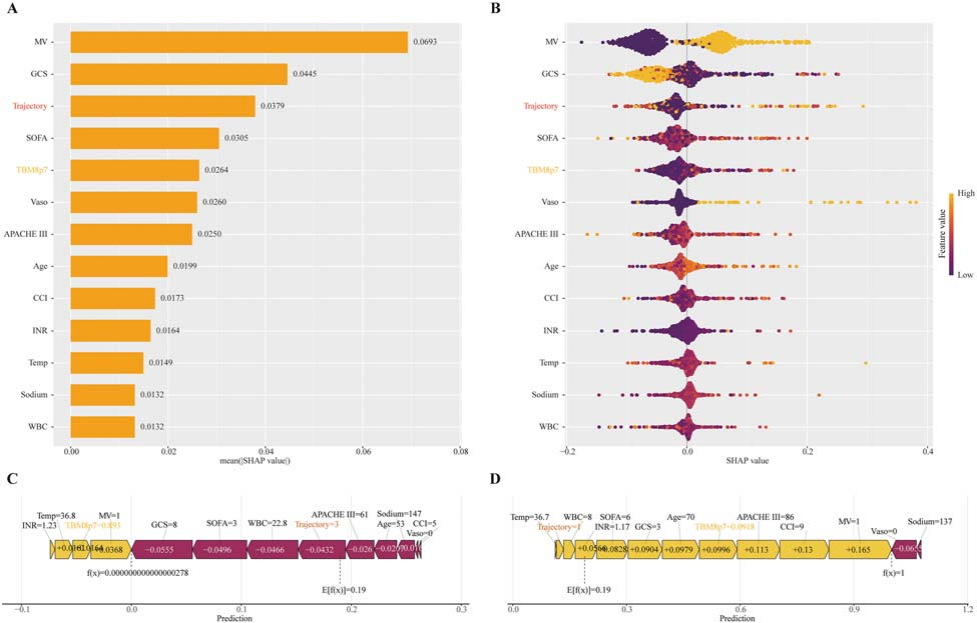
SHAP interpretation of the final extratrees classifier in the training set. SHAP was computed for the final ExtraTrees classifier fitted on the training set. **(A)** ranks global feature importance by mean absolute SHAP value. **(B)** (beeswarm) shows patient-level contributions (rightward values increase predicted in-hospital mortality; color encodes feature value from low to high). **(C–D)** are force plots for two representative patients, illustrating how each feature shifts the prediction from the baseline to the individual risk. Variables displayed are those retained for modeling: TyG trajectory and TBM8p7 (dynamic features), plus Age, Temperature, WBC, Sodium, INR, APACHE III, CCI, GCS, SOFA, vasopressor use, and mechanical ventilation.

SHAP was used to interpret the final ExtraTrees classifier, with global and patient-level explanations summarized in Figure 9. The global importance ranking highlighted MV and GCS as the dominant contributors, followed by TyG trajectory and TBM8p7 alongside SOFA and vasopressor use (Figures 9A–B). Case-level SHAP plots showed consistent directionality, with higher TyG trajectories and larger TBM8p7 values increasing predicted risk (Figures 9C–D). Complementary model-agnostic importance analyses (loss-based and permutation importance) supported a similar profile (Supplementary Figures 14, 15). The model was implemented as a web-based tool to return individualized risk estimates together with feature-level explanations (Supplementary Figure 16, https://njudrumtowernsicu.shinyapps.io/ABI_Prognosis_ModelTyG/).

## 4 Discussion

Our findings show that TyG needs to be viewed as a time-varying metabolic signal in ABI rather than a one-off laboratory value. We identified three stable trajectories that became prognostically relevant only after day 7, and a cumulative TyG burden (TBM8p7) that tracked mortality in a graded fashion. Adding these dynamic metrics to an ICU-feasible prediction model kept them among the most influential predictors, indicating real incremental information beyond conventional severity scores.

Most clinical reports on TyG in acute neurological or ICU populations have been admission-based: higher TyG at ICU entry or during the index ICU stay was associated with in-hospital or short-term mortality, and the association tended to be clearer in metabolically vulnerable stroke subgroups (19, 22, 23). However, an analysis that pooled two large cohorts and incorporated Mendelian-randomization instruments found an apparently protective association, underscoring how a single–time-point TyG measurement can change direction depending on timing and case mix (15). This suggests that baseline TyG does carry prognostic signal, but by design these studies cannot tell apart transient stress hypermetabolism from a sustained dysmetabolic state.

Longitudinal and burden-oriented investigations outside the ICU have started to make that distinction clearer. Cohorts that tracked changes in TyG showed that trajectories that failed to fall, or even rose, were the ones that translated into later cardiovascular or cerebrovascular events (24). Likewise, studies that summed cumulative exposure to elevated TyG demonstrated a graded relationship with downstream cardiometabolic outcomes, implying that duration and magnitude both matters (25). Similar separation of higher-risk metabolic phenotypes has also been reported for TyG-based composite or inflammatory–metabolic trajectories, such as TyG–CVAI and CRP–TyG patterns, supporting the idea that “staying high” is biologically different from “spiking once” (26, 27). What had been missing was a test of this principle in a multicenter ICU ABI population, where the stress response is sharper and laboratory sampling is denser.

Our ABI findings align with this time-sensitive view of TyG. ABI triggers a neuroendocrine stress response that makes glucose control difficult to individualize, and over-suppression has been cautioned against in recent ICU guidelines (5). Patients who can downregulate this response in the first several days manifested the LSI pattern we identified, whereas those with persistent neuroendocrine drive, pre-existing insulin resistance, inflammation, or ongoing organ/nutritional support remained in MI/PH patterns. The observation that all TBM strata were associated with mortality, and that TBM8p7 stayed significant after adjustment, mirrors what cumulative-exposure studies have shown and supports the interpretation that, in ABI, how long and how high the metabolic insult persists is more informative than a single elevated value (25).

In our cohort, TyG trajectories violated the proportional-hazards assumption in the unstratified Cox model, and the LSI, MI, and PH curves separated only after day 7, suggesting a delayed prognostic contribution of metabolic heterogeneity. This pattern is consistent with acute stroke data showing that mortality in the first week is driven mainly by initial neurological severity, overall clinical status, and adherence to early processes of care, leaving little residual variance for metabolic factors to explain (28–30). It is also in line with ICU practice, where a 7–10-day window is often used to distinguish patients who will recover promptly from those entering a prolonged, complication-prone course—for example, when evaluating early vs. late mobilization or determining tracheostomy timing, including in TBI cohorts (31–33). Together, these observations support that our 7-day split reflects a clinically meaningful inflection point rather than a modeling artifact: before day 7, deaths are predominantly brain-injury–driven; after day 7, outcomes increasingly depend on secondary complications and on the patient's ability to exit the stress/insulin-resistant state, which is precisely where PH trajectories and high TBM became independent signals in our analysis.

Recent ML work in acute stroke and general ICU populations has shown that models built on large sets of static admission variables often fail to outperform well-specified regression models, so we adopted a feature-first strategy instead (34–36). We first fixed a small, ICU-feasible consensus set of routinely collected predictors using complementary selection methods, and then we explicitly added the two time-dependent metabolic predictors that our survival analyses had identified as prognostic, namely TyG trajectory and TBM8p7, consistent with recent ML studies that treated TyG as a real signal in critically ill stroke patients (20). Using this fixed feature set, we benchmarked 14 classifiers and selected ExtraTrees because it showed the smallest internal-to-external performance drop; in other words, we prioritized transportability over maximizing apparent discrimination. The lower external AUROC likely reflects cross-database heterogeneity and distribution shift between NSICU and the pooled MIMIC-IV/eICU cohorts. Although AUROC is threshold-independent, differences in outcome prevalence and risk-score distributions may affect operating characteristics (e.g., sensitivity and specificity) when applying a prespecified threshold, underscoring the need for cohort-specific recalibration and prospective validation. SHAP and permutation importance showed that TyG trajectory and TBM8p7 sat in the same importance tier as neurological status, severity scores, and organ-support variables. This indicates that dynamic TyG load represents an independent prognostic stream rather than merely reflecting overall ICU severity, and that its value extends beyond admission-only models. Given the compact, interpretable predictor set, we implemented a web-based demonstrator to support transparency and reproducibility; cohort-specific recalibration and prospective validation are still needed for broader use.

### 4.1 Limitations

As a retrospective analysis of fixed databases, our findings are observational and cannot establish causality; unmeasured heterogeneity in case mix, sampling, and treatment practices may persist despite multivariable adjustment. Because ABI in this study was operationalized as stroke and TBI, the generalizability of our findings to other ABI entities (e.g., post–cardiac arrest hypoxic–ischemic brain injury) remains uncertain. Several ABI-specific clinical details (e.g., neuroimaging and lesion characteristics) and longer-term neurological outcomes were unavailable, which restricted us to in-hospital mortality. Model discrimination also attenuated on external validation, and operating-threshold selection (including Youden optimization) may shift the sensitivity–specificity balance, indicating that the approach needs confirmation in independent, prospectively collected ABI cohorts. The web tool is intended for transparency and reproducibility, and the TyG phenotypes are hypothesis-generating rather than prescriptive. Future work should prospectively test whether patients who remain in higher TyG trajectories after day 7 benefit from targeted metabolic or infection-control strategies.

## 5 Conclusion

In this multicenter ICU ABI cohort, TyG needed to be modeled as a time-aware metabolic exposure: dynamic TyG trajectories became prognostically informative only after day 7, and cumulative TyG burden (TBM8p7) showed a parallel, graded association with in-hospital mortality, indicating metabolic risk that is not captured by conventional severity scores.

## Data Availability

The datasets presented in this study can be found in online repositories. The names of the repository/repositories and accession number(s) can be found in the article/Supplementary material.
