# Supplementary material for: Dynamic TyG trajectories cumulative TyG burden are associated with in-hospital mortality in acute brain injury: a multicenter interpretable machine-learning analysis
Source: Front Nutr. 2026 Feb 27;13:1761240. doi: 10.3389/fnut.2026.1761240 (PMC12982064; doi:10.3389/fnut.2026.1761240)
Supplement: Supplementary file 1 [file Data_Sheet_1.pdf]

Supplementary Table 1. Clinical Variables Extracted for Analysis

Category	Variables
**Demographics & admission**	Age* (years); Sex (male/female); Body Mass Index (BMI)# (kg/m²); Trauma (yes/no); Cerebral Infarction (CI) (yes/no); Admission Type (AdmType) (elective/emergency); Admission Time (AdmTime) (day/night)
**Physiological parameters**	Temperature* (°C); Respiratory Rate* (RR) (breaths/min); Heart Rate* (HR) (bpm); Mean Blood Pressure* (MBP) (mmHg)
**Laboratory & derived measures**	Urine Output* (UO) (mL); Red Blood Cell count* (RBC) ($\times 10^{12}$/L); White Blood Cell count* (WBC) ($\times 10^{9}$/L); Platelet count* (PLT) ($\times 10^{9}$/L); Alanine Aminotransferase# (ALT) (U/L); Blood Urea Nitrogen# (BUN) (mg/dL); Creatinine# (Cr) (mg/dL); Glucose (mg/dL); Triglycerides (TG) (mg/dL); Triglyceride-glucose (TyG) Index*; Sodium* (mmol/L); Potassium* (mmol/L); International Normalized Ratio* (INR); Albumin* (g/L)
**Comorbidities & severity**	Hypertension (HTN) (yes/no); Diabetes Mellitus (DM) (yes/no); Chronic kidney disease (CKD) (yes/no); Liver Disease (LD) (yes/no); Charlson Comorbidity Index# (CCI); Sequential Organ Failure Assessment# (SOFA); Acute Physiology and Chronic Health Evaluation III* (APACHE III); Glasgow Coma Scale (GCS) at Admission
**Treatments**	Mechanical Ventilation (MV) (yes/no); Vasopressor Use (Vaso) (yes/no); Mannitol (yes/no); Sedation (yes/no); Dialysis (yes/no); Craniotomy (yes/no); Embolization (yes/no); Intracranial Pressure Monitoring (ICP) (yes/no)
**Discharge disposition**	Discharge location (acute/ICU care; chronic/long-term care; home care/recovery)
**Outcomes & length of stay**	Hospital Length of Stay (LOS) # (days); ICU Length of Stay (ICU LOS) # (days); In-Hospital Mortality (yes/no)

Notes: * continuous (approximately normal), reported as mean ± SD; # continuous (skewed), reported as median (IQR); variables without symbols are categorical and reported as n (%); TyG formula: $TyG = \ln(\text{Triglycerides [mg/dL]} \times \text{Glucose [mg/dL]} / 2)$ (original and z-score; dimensionless); medications recorded within the first 24 hours after ICU admission included vasopressors, mannitol, and sedatives. Abbreviations: ALT, Alanine Aminotransferase; APACHE III, Acute Physiology and Chronic Health Evaluation III; BMI, Body Mass Index; BUN, Blood Urea Nitrogen; CCI, Charlson Comorbidity Index; CI, Cerebral Infarction; CKD, Chronic Kidney Disease; Cr, Creatinine; DM, Diabetes Mellitus; GCS, Glasgow Coma Scale; HR, Heart Rate; HTN, Hypertension; ICP, Intracranial Pressure; ICU LOS, Intensive Care Unit Length of Stay; INR, International Normalized Ratio; LD, Liver Disease; LOS, Length of Stay; MBP, Mean Blood Pressure; MV, Mechanical Ventilation; PLT, Platelet count; RBC, Red Blood Cell count; RR, Respiratory Rate; SOFA, Sequential Organ Failure Assessment; TG, Triglycerides; TyG, Triglyceride-

glucose; UO, Urine Output; Vaso, Vasopressor; WBC, White Blood Cell count.

Supplementary Table 2. Missingness of Baseline Covariates in the Integrated Cohort

Variable	Missing, n	Missing, %
**Urine output, mL**	622	13.07
**BMI, kg/m²**	1086	22.82
**ALT, U/L**	236	4.96
**GCS at admission**	113	2.37
**INR**	108	2.27
**WBC**	98	2.06
**BUN, mg/dL**	78	1.64
**Creatinine, mg/dL**	75	1.58
**Sodium, mmol/L**	72	1.51
**Temperature, °C**	39	0.82
**SOFA**	80	1.68
**Albumin, g/L**	459	9.64
**Respiratory rate, breaths/min**	16	0.34
**RBC, ×10^{12}/L**	14	0.29
**MBP, mmHg**	13	0.27
**PLT, ×10^{9}/L**	10	0.21
**Heart rate, bpm**	8	0.17
**Potassium, mmol/L**	7	0.15

Notes: Values are shown as n (%) with missing data for each baseline covariate before imputation; covariates not listed had 0% missingness.

Supplementary Figure 1. Observed vs Imputed-Only Distributions for Key Covariates

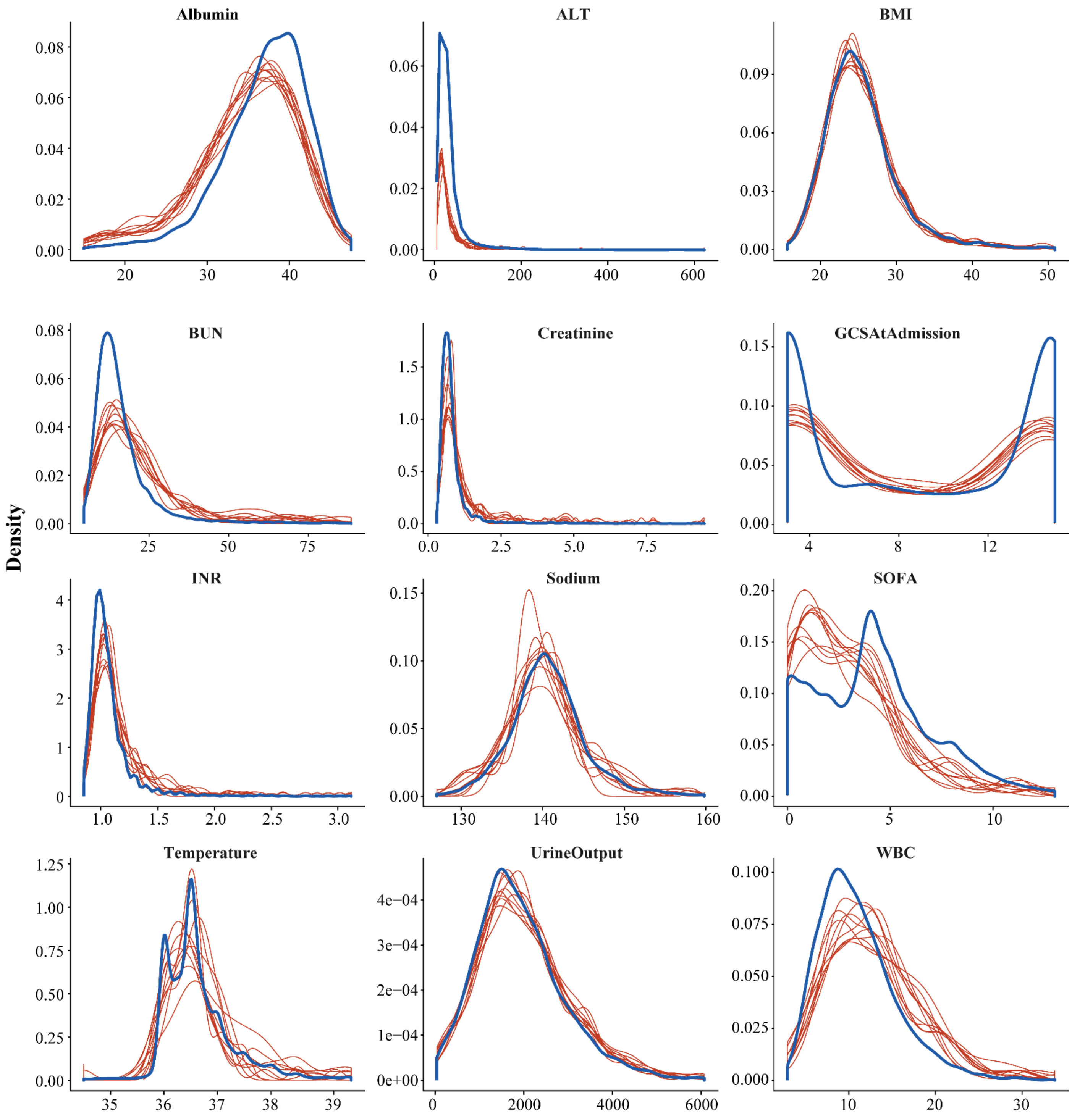

Legend: The thick blue line shows the distribution of observed (non-missing) values, and the thin red lines show the distributions of imputed values for missing entries only across 10 imputations (m = 10). Distributions are presented as kernel density estimates for each covariate.

Supplementary Figure 2. Observed vs Completed-Data Distributions for Key Covariates

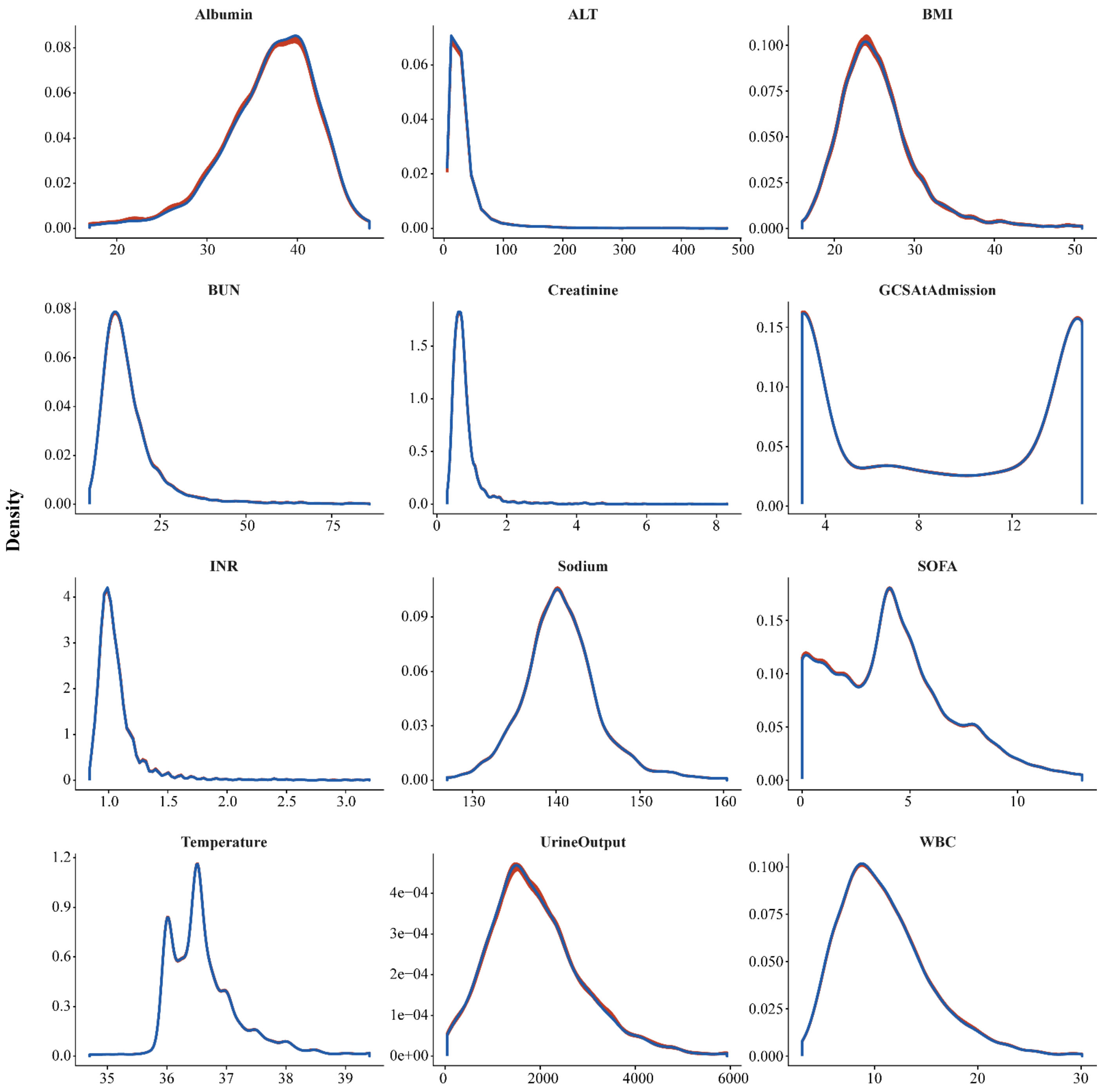

Legend: The thick blue line shows the distribution of observed (non-missing) values, and the thin red lines show the distributions of each completed dataset (observed + imputed) across 10 imputations (m = 10). Distributions are presented as kernel density estimates for each covariate.

Supplementary Figure 3. TyG measurement accumulation and timing by trajectory class and survival status

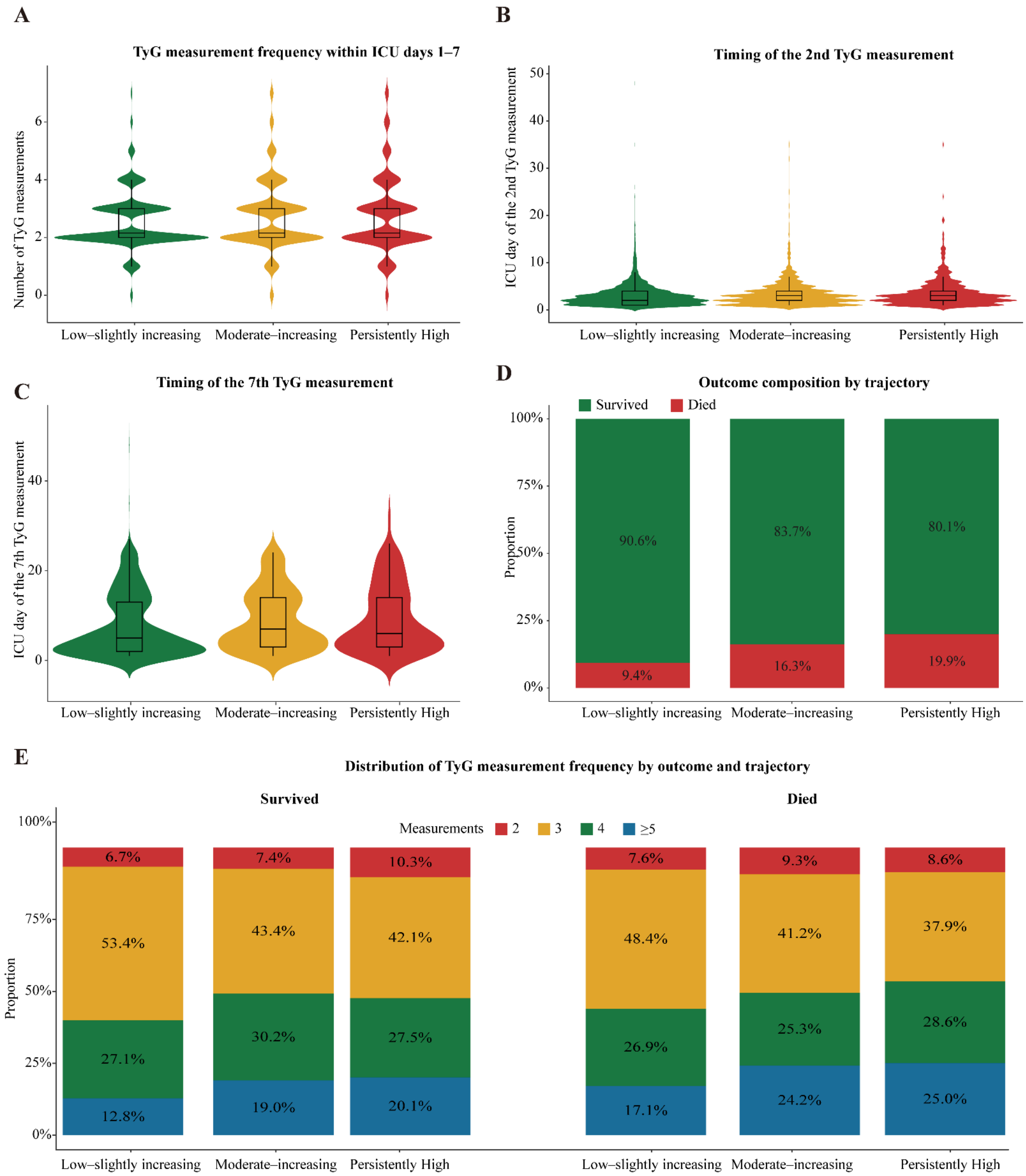

Legend: Shown are the number and timing of recorded TyG measurements by trajectory class and survival status, including the distribution of measurement counts within ICU days 1–7 (A), the ICU day of the second measurement (B), the ICU day of the seventh measurement among patients who reached seven

measurements (C), the outcome composition within each class (D), and the distribution of measurement-count categories within ICU days 1–7 stratified by survival status (E).

Supplementary Figure 4. Threshold-Based Mean AUC (TBM) for TyG

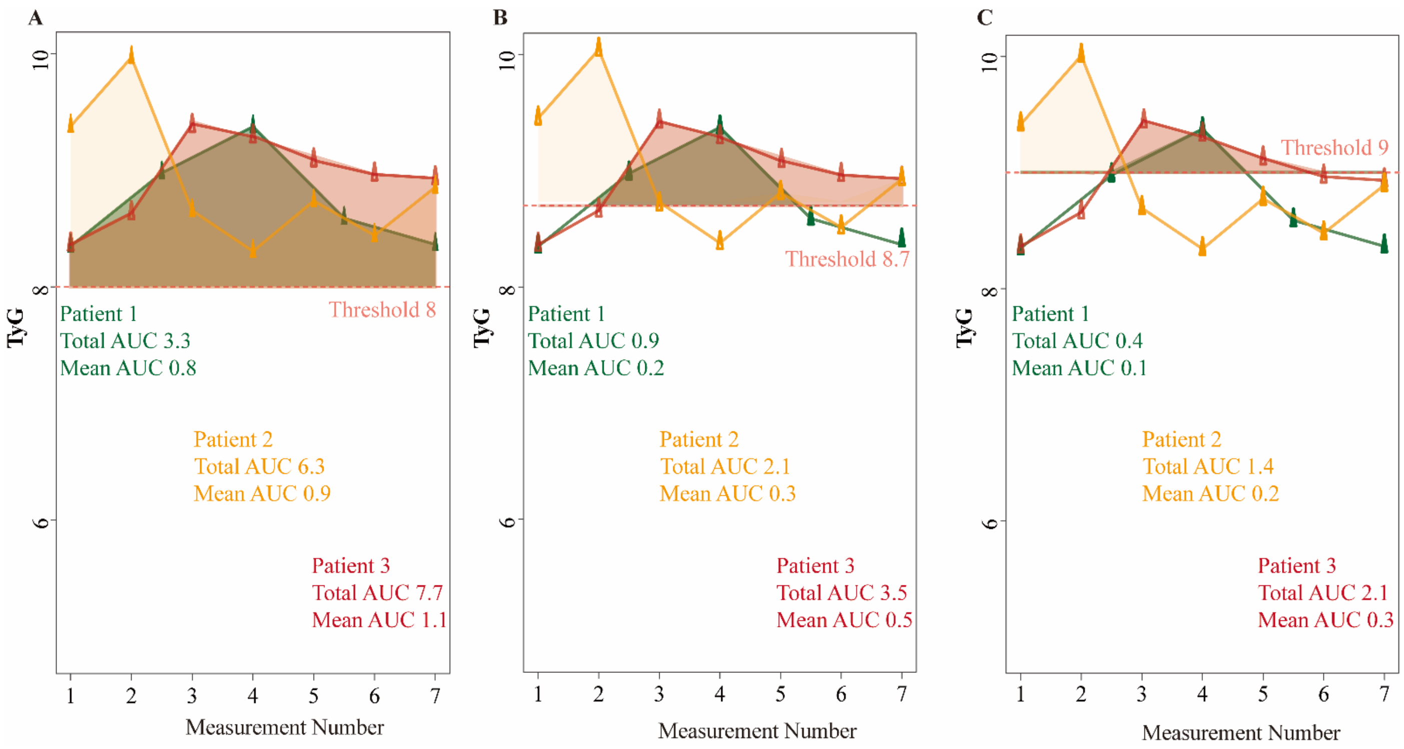

Legend: Panels A–C illustrate TBM computation for three example patients at TyG thresholds of 8.0, 8.7, and 9.0. The dashed horizontal line marks the threshold; lines show serial TyG values. Shaded regions represent the zero-truncated trapezoidal integral of supra-threshold TyG ("Total AUC") across adjacent measurement intervals. Mean AUC (TBM) is defined as Total AUC divided by the number of observed intervals (N−1), where N is the number of TyG measurements available for that patient. Higher TBM denotes greater cumulative supra-threshold TyG exposure.

Supplementary Table 3. Model Fit Indices for TyG Trajectories in the Integrated Cohort

G	Log-Likelihood	AIC	BIC	SABIC	Entropy	Class Proportion (%)						Average Posterior Probability					
						1	2	3	4	5	6	1	2	3	4	5	6
2	-20102.01	40234.03	40331.05	40283.38	0.27	52.58	47.42					0.69	0.80				
3	-20094.65	40229.30	40358.66	40295.11	0.26	61.76	23.47	14.77				0.69	0.50	0.63			
4	-20081.89	40213.79	40375.49	40296.05	0.42	21.28	44.66	33.57	0.48			0.64	0.57	0.73	0.55		
5	-20080.72	40221.44	40415.48	40320.15	0.34	30.78	23.19	7.00	37.35	1.68		0.62	0.41	0.37	0.59	0.54	
6	-20062.74	40195.48	40421.86	40310.64	0.52	17.31	56.64	3.40	12.86	3.17	6.62	0.69	0.71	0.44	0.54	0.63	0.67

Note: Latent class growth modeling (LCGM) was applied to standardized serial TyG measurements in the integrated cohort (NSICU, MIMIC-IV, and eICU combined). Fit indices were log-likelihood, AIC, BIC, and SABIC; classification quality was summarized by entropy, class proportions (%), and average posterior probabilities (APP).

Supplementary Table 4. Model Fit Indices for TyG Trajectories in the NSICU Cohort

G	Log-Likelihood	AIC	BIC	SABIC	Entropy	Class Proportion (%)						Average Posterior Probability					
						1	2	3	4	5	6	1	2	3	4	5	6
2	-9789.70	19609.41	19703.13	19655.46	0.27	58.76	41.24					0.71	0.80				
3	-9780.13	19600.26	19725.22	19661.67	0.31	65.91	24.19	9.90				0.73	0.53	0.67			
4	-9764.56	19579.13	19735.32	19655.88	0.44	20.01	54.70	23.23	2.07			0.69	0.64	0.70	0.56		
5	-9761.40	19582.79	19770.22	19674.90	0.50	21.81	54.20	0.42	19.14	4.43		0.71	0.64	0.46	0.66	0.54	
6	-9759.39	19588.78	19807.45	19696.23	0.44	1.99	1.54	30.14	40.30	22.68	3.35	0.40	0.46	0.64	0.53	0.66	0.57

Note: LCGM was applied to standardized serial TyG measurements in NSICU. Fit indices included log-likelihood, AIC, BIC, and SABIC; classification quality was summarized by entropy, class proportions (%), and APP.

Supplementary Table 5. Model Fit Indices for TyG Trajectories in the MIMIC-IV Cohort

G	Log-Likelihood	AIC	BIC	SABIC	Entropy	Class Proportion (%)						Average Posterior Probability					
						1	2	3	4	5	6	1	2	3	4	5	6
2	-1025.89	2081.78	2138.72	2091.14	0.30	71.73	28.27					0.74	0.83				
3	-992.72	2025.43	2101.36	2037.92	0.90	87.23	8.51	4.26				0.98	0.80	0.87			
4	-1006.98	2063.96	2158.86	2079.56	0.58	20.67	38.91	17.93	22.49			0.83	0.69	0.89	0.73		
5	-1002.38	2064.76	2178.64	2083.48	0.59	19.76	37.69	8.21	16.11	18.24		0.83	0.69	0.57	0.87	0.64	
6	-993.39	2056.78	2189.64	2078.62	0.63	17.93	39.82	10.64	7.60	5.78	18.24	0.84	0.73	0.61	0.78	0.78	0.66

Note: LCGM was applied to standardized serial TyG in MIMIC-IV; reported metrics include model fit (log-likelihood, AIC, BIC, SABIC) and classification (entropy, class proportions, APP).

Supplementary Table 6. Model Fit Indices for TyG Trajectories in the eICU Cohort

G	Log-Likelihood	AIC	BIC	SABIC	Entropy	Class Proportion (%)						Average Posterior Probability					
						1	2	3	4	5	6	1	2	3	4	5	6
2	-1394.16	2818.32	2884.57	2836.95	0.47	53.43	46.57					0.82	0.84				
3	-1390.84	2821.69	2910.02	2846.52	0.40	54.08	25.49	20.42				0.78	0.54	0.68			
4	-1373.36	2796.71	2907.13	2827.76	0.71	28.43	55.72	8.50	7.35			0.85	0.85	0.65	0.75		
5	-1371.27	2802.54	2935.04	2839.80	0.53	29.08	20.92	32.52	8.66	8.82		0.73	0.57	0.67	0.66	0.79	
6	-1364.20	2798.41	2952.99	2841.87	0.56	10.95	42.65	5.72	28.27	9.64	2.78	0.67	0.68	0.54	0.61	0.78	0.63

Note: LCGM was applied to standardized serial TyG measurements in eICU. Fit indices included log-likelihood, AIC, BIC, and SABIC; classification quality was summarized by entropy, class proportions (%), and APP.

Supplementary Figure 5. TyG Trajectory Phenotypes in the NSICU Cohort

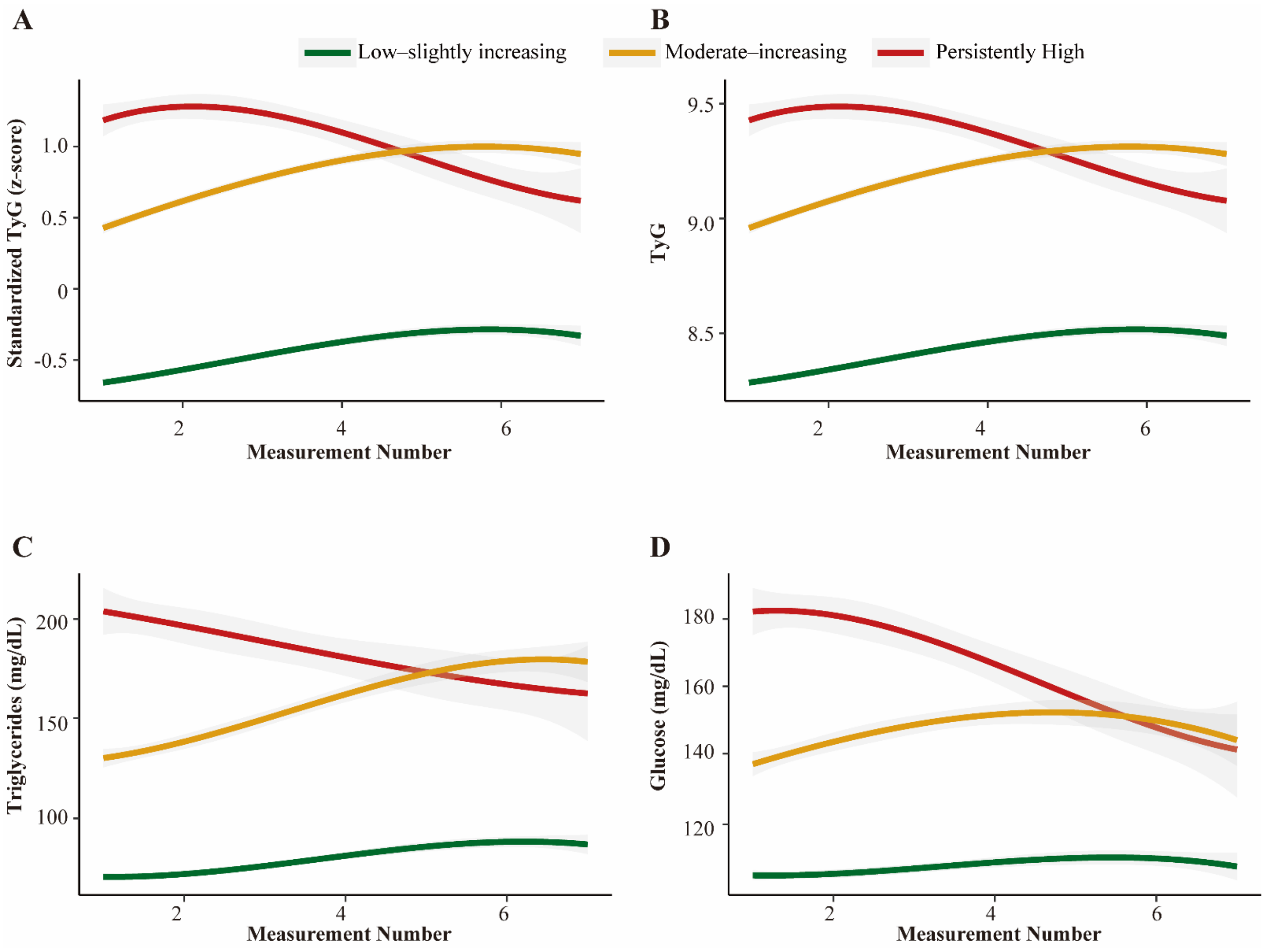

Legend: Panels A–D show standardized TyG z-score, TyG index, triglycerides (mg/dL), and glucose (mg/dL) by measurement number (1–7) in the NSICU cohort. Lines denote model-estimated means; shaded areas indicate 95% confidence intervals. Colors denote trajectories: green, Low–slightly increasing (LSI); yellow, Moderate–increasing (MI); red, Persistently High (PH).

Supplementary Figure 6. TyG Trajectory Phenotypes in the MIMIC-IV Cohort

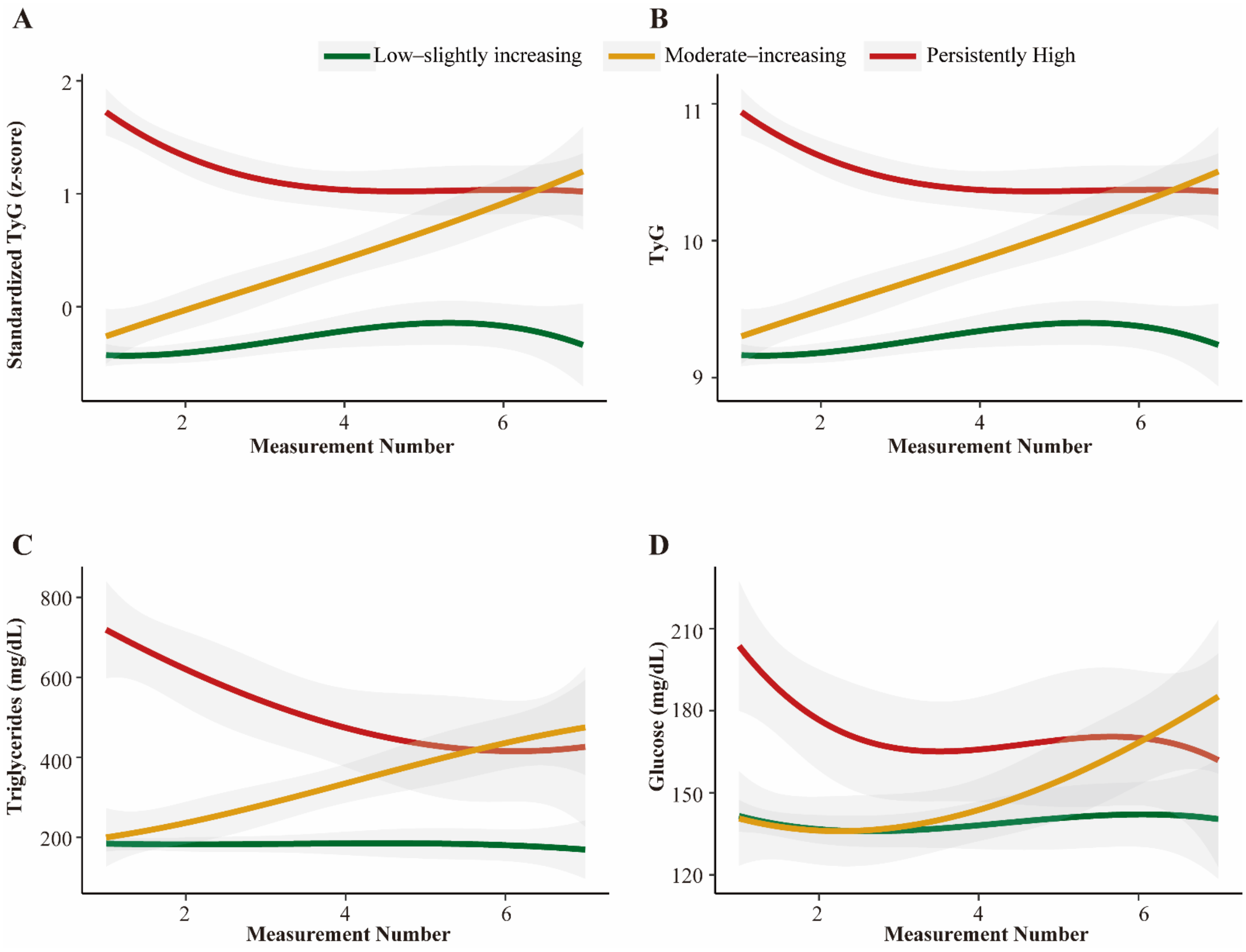

Legend: Panels A–D show standardized TyG z-score, TyG index, triglycerides (mg/dL), and glucose (mg/dL) by measurement number (1–7) in the MIMIC-IV cohort. Lines denote model-estimated means; shaded areas indicate 95% confidence intervals. Colors denote trajectories: green, Low–slightly increasing (LSI); yellow, Moderate–increasing (MI); red, Persistently High (PH).

Supplementary Figure 7. TyG Trajectory Phenotypes in the eICU Cohort

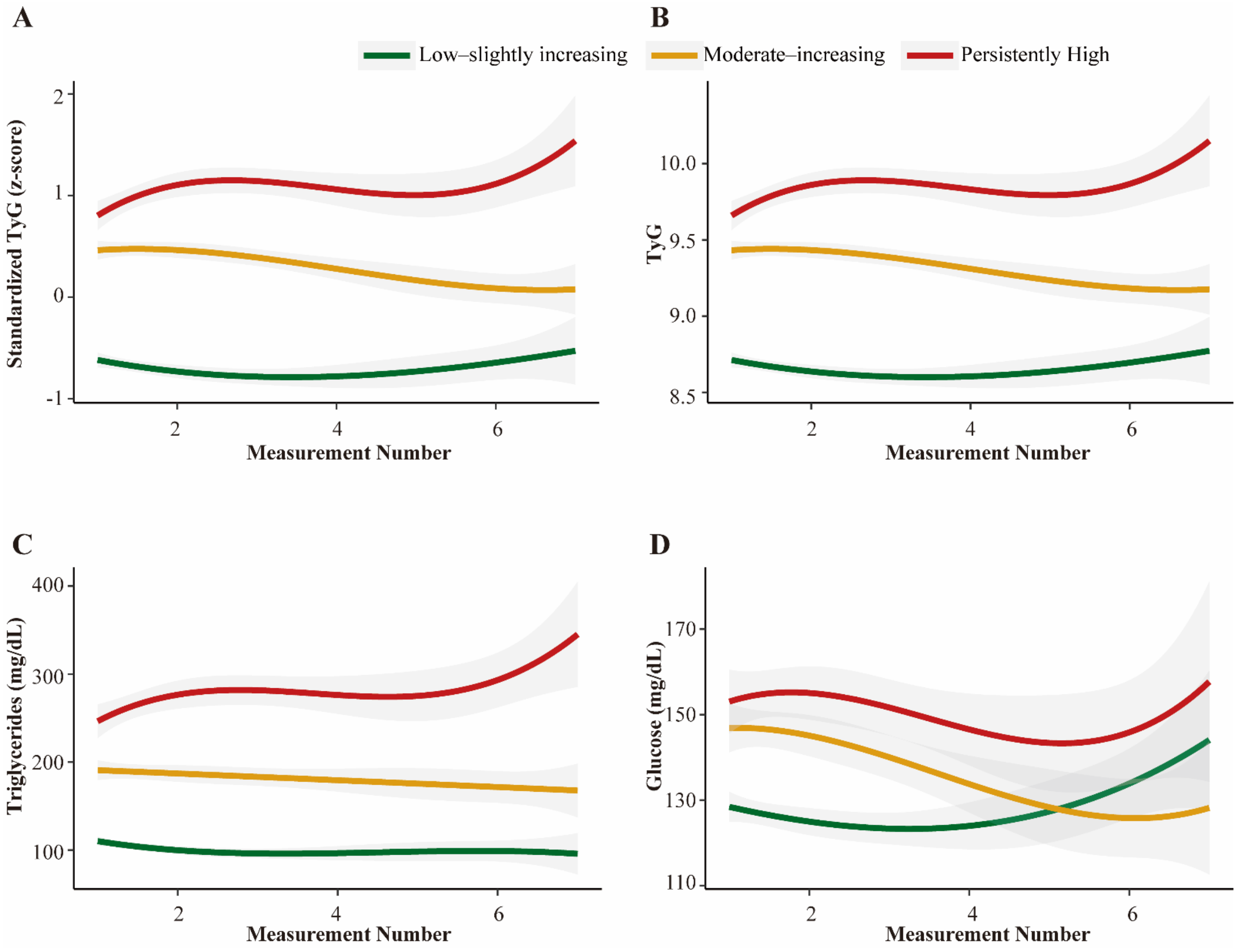

Legend: Panels A–D show standardized TyG z-score, TyG index, triglycerides (mg/dL), and glucose (mg/dL) by measurement number (1–7) in the eICU cohort. Lines denote model-estimated means; shaded areas indicate 95% confidence intervals. Colors denote trajectories: green, Low–slightly increasing (LSI); yellow, Moderate–increasing (MI); red, Persistently High (PH).

Supplementary Figure 8. TyG Trajectory Phenotypes Based on 10 Sequential Measurements

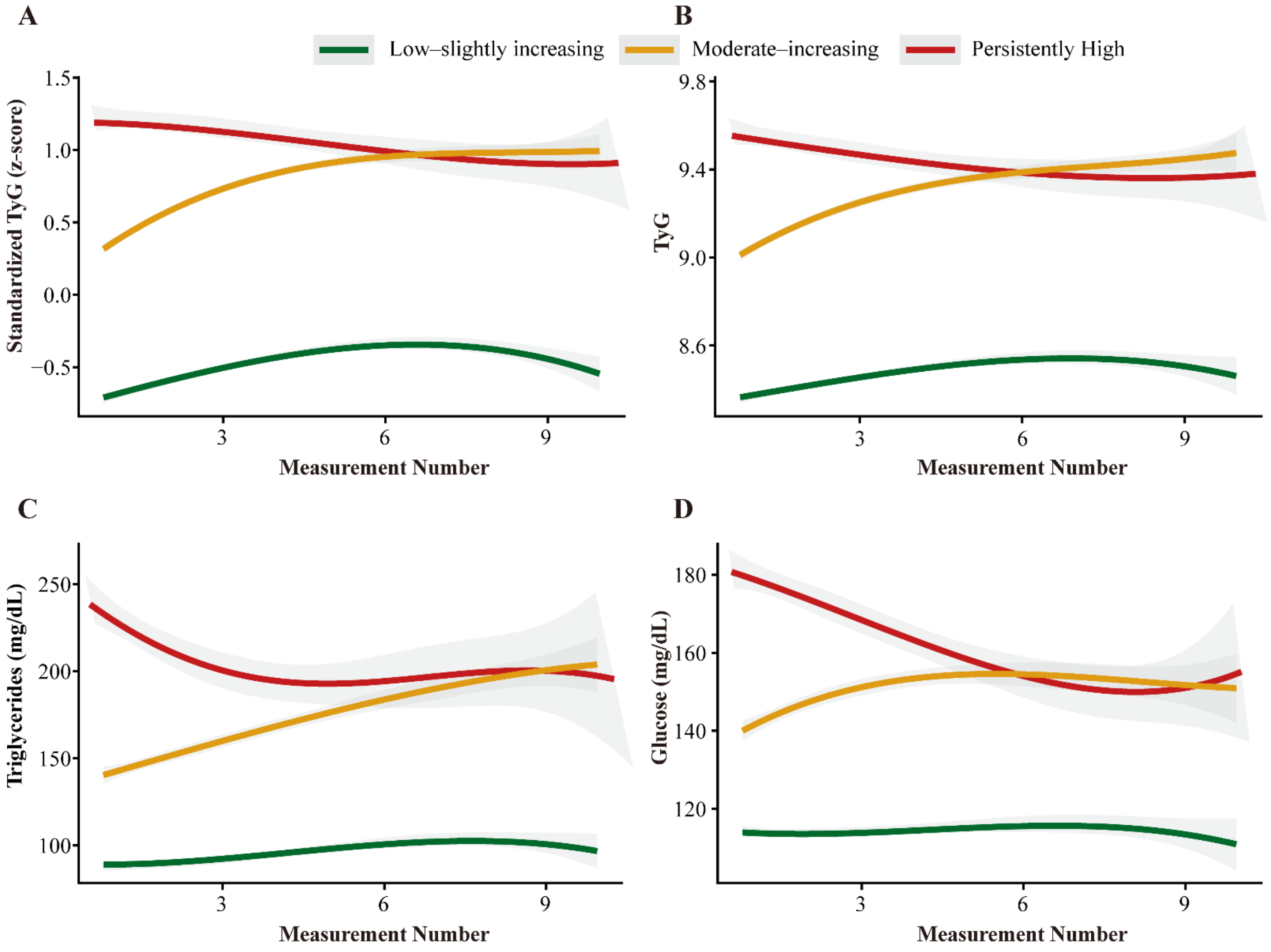

Legend: Panels A–D show standardized TyG z-score, TyG index, triglycerides (mg/dL), and glucose (mg/dL) by measurement number (1–10) in the integrated cohort. Uncertainty increases at later measurement numbers because fewer measurements were available, resulting in wider 95% confidence intervals.

Supplementary Table 7. Additional Baseline Characteristics by TyG Trajectory

Characteristics	Total (n = 4760)	Low–slightly increasing (n = 2940)	Moderate–increasing (n = 1117)	Persistently High (n = 703)	*P*
**Demographics & admission**					
**Admission Type**	3184 (66.9)	1914 (65.1)	780 (69.8)	490 (69.7)	0.004
**Admission Time**					0.002
**Day Shift**	2838 (59.6)	1803 (61.3)	653 (58.5)	382 (54.3)	
**Night Shift**	1922 (40.4)	1137 (38.7)	464 (41.5)	321 (45.7)	
**CI**	751 (15.8)	469 (16.0)	161 (14.4)	121 (17.2)	0.257
**Physiological & laboratory**					
**Temperature (°C)**	36.6 ± 0.7	36.6 ± 0.6	36.7 ± 0.7	36.8 ± 0.9	<0.001
**RR (breaths/min)**	19.0 ± 7.0	18.8 ± 6.7	19.1 ± 7.1	19.7 ± 7.9	0.009
**MBP (mmHg)**	98.9 ± 21.1	98.0 ± 20.1	100.5 ± 22.1	99.7 ± 23.7	0.003
**UO (mL)**	1931.5 ± 1052.2	1912.7 ± 1002.6	1954.5 ± 1142.5	1972.1 ± 1098.2	0.335
**RBC ($\times 10^{12}$/L)**	4.0 ± 0.7	4.0 ± 0.7	4.0 ± 0.7	3.9 ± 0.8	0.096
**WBC ($\times 10^{9}$/L)**	11.1 ± 5.0	10.4 ± 4.5	12.0 ± 5.0	12.2 ± 6.2	<0.001
**PLT ($\times 10^{9}$/L)**	186.7 ± 75.5	186.8 ± 75.6	188.4 ± 72.2	183.6 ± 80.3	0.406
**ALT (U/L)**	18.4 (13.0, 29.3)	17.1 (12.3, 27.0)	20.7 (14.2, 32.0)	21.6 (14.6, 36.1)	<0.001
**Albumin (g/L)**	37.1 ± 5.3	37.3 ± 5.1	37.1 ± 5.3	36.3 ± 5.9	<0.001
**Potassium (mmol/L)**	3.8 ± 0.5	3.8 ± 0.4	3.8 ± 0.5	3.8 ± 0.5	0.413
**INR**	1.1 ± 0.4	1.1 ± 0.4	1.1 ± 0.3	1.1 ± 0.4	0.070
**Comorbidities**					
**Liver Disease**	1043 (21.9)	551 (18.7)	291 (26.1)	201 (28.6)	<0.001
**Severity & ICU interventions**					
**Dialysis**	112 (2.4)	42 (1.4)	31 (2.8)	39 (5.5)	<0.001
**ICP monitoring**	1008 (21.2)	530 (18.0)	297 (26.6)	181 (25.7)	<0.001
**Mannitol**	1423 (29.9)	769 (26.2)	386 (34.6)	268 (38.1)	<0.001
**Sedation**	1889 (39.7)	1002 (34.1)	532 (47.6)	355 (50.5)	<0.001
**Outcomes**					
**Discharge Location**					<0.001
**Acute/ICU**	1602 (33.7)	775 (26.4)	484 (43.3)	343 (48.8)	
**Chronic/LTC**	1280 (26.9)	777 (26.4)	313 (28.0)	190 (27.0)	

**Home Care**	1878 (39.5)	1388 (47.2)	320 (28.6)	170 (24.2)

Notes: Continuous variables are summarized as mean ± SD or median (IQR); categorical variables as n (%). For binary variables, n (%) indicates the presence of the characteristic (Yes). Abbreviations: CI, cerebral infarction; RR, respiratory rate; MBP, mean blood pressure; UO, urine output; RBC, red blood cell count; WBC, white blood cell count; PLT, platelet count; ALT, alanine aminotransferase; INR, international normalized ratio; ICP, intracranial pressure monitoring; LTC, long-term care.

Supplementary Table 8. Covariate Screening for Multivariable Models Using CIE and Collinearity Diagnostics

Crude Model			Full Model			VIF	Select	Select VIF
**Term1**	**Coeff1**	**Change1 (%)**	**Term2**	**Coeff2**	**Change2 (%)**			
Crude for Trajectory = 2	0.41	Ref.	Full for Trajectory = 2	0.23	Ref.	1.277	Ref.	Ref.
Crude for Trajectory = 3	0.52	Ref.	Full for Trajectory = 3	0.27	Ref.	1.277	Ref.	Ref.
Age for Trajectory = 2	0.44	5.8	Age for Trajectory = 2	0.21	-7.6	2.275	No	No
Age for Trajectory = 3	0.59	13.3	Age for Trajectory = 3	0.20	-25	2.275	Yes	Yes
Sex for Trajectory = 2	0.41	-0.1	Sex for Trajectory = 2	0.23	1.2	1.179	No	No
Sex for Trajectory = 3	0.52	-0.2	Sex for Trajectory = 3	0.27	1.2	1.179	No	No
BMI for Trajectory = 2	0.42	0.4	BMI for Trajectory = 2	0.23	0.1	1.080	No	No
BMI for Trajectory = 3	0.53	0.3	BMI for Trajectory = 3	0.27	0.5	1.080	No	No
Admission Type for Trajectory = 2	0.41	-1.2	Admission Type for Trajectory = 2	0.23	-0.3	1.124	No	No
Admission Type for Trajectory = 3	0.52	-0.7	Admission Type for Trajectory = 3	0.27	0.3	1.124	No	No
Admission Time for Trajectory = 2	0.42	0.8	Admission Time for Trajectory = 2	0.23	-0.7	1.150	No	No
Admission Time for Trajectory = 3	0.52	-0.5	Admission Time for Trajectory = 3	0.27	0.0	1.150	No	No
Trauma for Trajectory = 2	0.41	-1.7	Trauma for Trajectory = 2	0.25	7.4	1.356	No	No
Trauma for Trajectory = 3	0.52	-0.3	Trauma for Trajectory = 3	0.29	7.0	1.356	No	No
Cerebral Infarction for Trajectory = 2	0.42	1.1	Cerebral Infarction for Trajectory = 2	0.22	-3.1	1.263	No	No
Cerebral Infarction for Trajectory = 3	0.52	-1.2	Cerebral Infarction for Trajectory = 3	0.27	0.2	1.263	No	No

Temperature for Trajectory = 2	0.41	-0.2	Temperature for Trajectory = 2	0.23	1.0	1.218	No	No
Temperature for Trajectory = 3	0.52	-0.2	Temperature for Trajectory = 3	0.27	0.9	1.218	No	No
Respiratory Rate for Trajectory = 2	0.41	-1.9	Respiratory Rate for Trajectory = 2	0.23	-1.1	1.520	No	No
Respiratory Rate for Trajectory = 3	0.5	-4.6	Respiratory Rate for Trajectory = 3	0.27	0.5	1.520	No	No
HR for Trajectory = 2	0.38	-6.9	HR for Trajectory = 2	0.24	2.5	1.702	No	No
HR for Trajectory = 3	0.45	-13.8	HR for Trajectory = 3	0.28	3.8	1.702	Yes	Yes
MBP for Trajectory = 2	0.41	0.3	MBP for Trajectory = 2	0.23	-0.9	1.222	No	No
MBP for Trajectory = 3	0.52	0.1	MBP for Trajectory = 3	0.27	0.3	1.222	No	No
Urine Output for Trajectory = 2	0.41	-0.1	Urine Output for Trajectory = 2	0.24	4.9	1.265	No	No
Urine Output for Trajectory = 3	0.52	-0.2	Urine Output for Trajectory = 3	0.29	7.1	1.265	No	No
RBC for Trajectory = 2	0.44	5.6	RBC for Trajectory = 2	0.23	0.0	1.722	No	No
RBC for Trajectory = 3	0.52	-1.3	RBC for Trajectory = 3	0.27	0.0	1.722	No	No
WBC for Trajectory = 2	0.38	-7.9	WBC for Trajectory = 2	0.25	6.1	1.355	No	No
WBC for Trajectory = 3	0.47	-9.8	WBC for Trajectory = 3	0.28	6.6	1.355	No	No
PLT for Trajectory = 2	0.42	1.8	PLT for Trajectory = 2	0.23	0.4	1.409	No	No
PLT for Trajectory = 3	0.51	-2	PLT for Trajectory = 3	0.27	-0.2	1.409	No	No
ALT for Trajectory = 2	0.41	-0.2	ALT for Trajectory = 2	0.23	-2.8	1.191	No	No
ALT for Trajectory = 3	0.52	-0.2	ALT for Trajectory = 3	0.26	-4.2	1.191	No	No
BUN for Trajectory = 2	0.36	-12.4	BUN for Trajectory = 2	0.23	0.4	2.804	Yes	Yes
BUN for Trajectory = 3	0.46	-12.2	BUN for Trajectory = 3	0.27	1.5	2.804	Yes	Yes
Albumin for Trajectory = 2	0.4	-2.4	Albumin for Trajectory = 2	0.23	-1.2	1.656	No	No
Albumin for Trajectory = 3	0.49	-7.3	Albumin for Trajectory = 3	0.27	-0.7	1.656	No	No

Creatinine for Trajectory = 2	0.38	-9.2	Creatinine for Trajectory = 2	0.24	2.1	2.587	No	No
Creatinine for Trajectory = 3	0.47	-10.5	Creatinine for Trajectory = 3	0.28	3.2	2.587	Yes	Yes
Sodium for Trajectory = 2	0.36	-11.8	Sodium for Trajectory = 2	0.25	9.8	1.252	Yes	Yes
Sodium for Trajectory = 3	0.48	-8.2	Sodium for Trajectory = 3	0.28	4.4	1.252	No	No
Potassium for Trajectory = 2	0.41	0.1	Potassium for Trajectory = 2	0.23	0.6	1.191	No	No
Potassium for Trajectory = 3	0.52	-1.0	Potassium for Trajectory = 3	0.27	0.4	1.191	No	No
INR for Trajectory = 2	0.42	2.5	INR for Trajectory = 2	0.23	-1.2	1.433	No	No
INR for Trajectory = 3	0.51	-2.6	INR for Trajectory = 3	0.27	-0.5	1.433	No	No
HTN for Trajectory = 2	0.4	-4.3	HTN for Trajectory = 2	0.23	-0.9	1.327	No	No
HTN for Trajectory = 3	0.5	-4.1	HTN for Trajectory = 3	0.26	-1.9	1.327	No	No
DM for Trajectory = 2	0.38	-6.9	DM for Trajectory = 2	0.22	-5.8	1.446	No	No
DM for Trajectory = 3	0.48	-8.9	DM for Trajectory = 3	0.25	-8.3	1.446	No	No
CKD for Trajectory = 2	0.37	-10.2	CKD for Trajectory = 2	0.23	0.4	1.816	Yes	Yes
CKD for Trajectory = 3	0.47	-10.6	CKD for Trajectory = 3	0.27	0.1	1.816	Yes	Yes
Liver Disease for Trajectory = 2	0.42	0.5	Liver Disease for Trajectory = 2	0.23	-1.4	1.187	No	No
Liver Disease for Trajectory = 3	0.53	0.5	Liver Disease for Trajectory = 3	0.26	-2.3	1.187	No	No
CCI for Trajectory = 2	0.37	-11.4	CCI for Trajectory = 2	0.23	-1.6	3.558	Yes	Yes
CCI for Trajectory = 3	0.45	-13.4	CCI for Trajectory = 3	0.27	0.7	3.558	Yes	Yes
SOFA for Trajectory = 2	0.33	-19.3	SOFA for Trajectory = 2	0.24	5.0	2.482	Yes	Yes
SOFA for Trajectory = 3	0.37	-29.1	SOFA for Trajectory = 3	0.27	2.9	2.482	Yes	Yes
APACHE III for Trajectory = 2	0.35	-15.3	APACHE III for Trajectory = 2	0.23	1.4	2.548	Yes	Yes

APACHE III for Trajectory = 3	0.41	-22.3	APACHE III for Trajectory = 3	0.27	1.0	2.548	Yes	Yes
GCS at admission for Trajectory = 2	0.3	-26.7	GCS at admission for Trajectory = 2	0.25	9.7	1.936	Yes	Yes
GCS at admission for Trajectory = 3	0.39	-25.6	GCS at admission for Trajectory = 3	0.30	10.9	1.936	Yes	Yes
MV for Trajectory = 2	0.30	-27.1	MV for Trajectory = 2	0.23	-0.2	1.292	Yes	Yes
MV for Trajectory = 3	0.37	-28.7	MV for Trajectory = 3	0.30	10.5	1.292	Yes	Yes
Dialysis for Trajectory = 2	0.39	-5.2	Dialysis for Trajectory = 2	0.23	-2.1	1.857	No	No
Dialysis for Trajectory = 3	0.47	-9.7	Dialysis for Trajectory = 3	0.26	-2.8	1.857	No	No
Craniotomy for Trajectory = 2	0.42	1.0	Craniotomy for Trajectory = 2	0.23	-1.7	1.501	No	No
Craniotomy for Trajectory = 3	0.52	0.1	Craniotomy for Trajectory = 3	0.27	0.0	1.501	No	No
ICP monitoring for Trajectory = 2	0.40	-3.8	ICP monitoring for Trajectory = 2	0.24	1.7	1.262	No	No
ICP monitoring for Trajectory = 3	0.52	-1.1	ICP monitoring for Trajectory = 3	0.26	-1.5	1.262	No	No
Embolization for Trajectory = 2	0.37	-9.8	Embolization for Trajectory = 2	0.24	2.5	1.228	No	No
Embolization for Trajectory = 3	0.44	-16.8	Embolization for Trajectory = 3	0.29	9.2	1.228	Yes	Yes
Vaso for Trajectory = 2	0.40	-3.5	Vaso for Trajectory = 2	0.22	-4.6	1.674	No	No
Vaso for Trajectory = 3	0.49	-5.6	Vaso for Trajectory = 3	0.24	-10.2	1.674	Yes	Yes
Mannitol for Trajectory = 2	0.40	-2.7	Mannitol for Trajectory = 2	0.23	-1.6	1.332	No	No
Mannitol for Trajectory = 3	0.50	-3.9	Mannitol for Trajectory = 3	0.28	3.3	1.332	No	No

Sedation for Trajectory = 2	0.41	0.3	Sedation for Trajectory = 2	0.23	1.2	1.177	No	No
Sedation for Trajectory = 3	0.53	0.3	Sedation for Trajectory = 3	0.27	1.2	1.177	No	No

Notes: Candidate covariates were screened by change-in-estimate (CIE), retaining variables that changed the trajectory effect by ≥10% in either contrast, with collinearity assessed using variance inflation factors (VIF). Trajectory 2 corresponds to the Moderate–increasing TyG class (MI) and Trajectory 3 to the Persistently High class (PH), each compared with Trajectory 1 (Low–slightly increasing) as the reference. In the table, Term1/Coeff1/Change1 (%) summarize the crude trajectory estimate and its percent change after adding each covariate; Term2/Coeff2/Change2 (%) report the corresponding results in the fully adjusted framework. Covariates retained by CIE and VIF screening were Age (age), Heart Rate (HR), Blood Urea Nitrogen (BUN), Creatinine (Cr), Sodium (sodium), chronic kidney disease (CKD), Charlson Comorbidity Index (CCI), Sequential Organ Failure Assessment (SOFA), Acute Physiology and Chronic Health Evaluation III (APACHE III), Glasgow Coma Scale at admission (GCS), Mechanical Ventilation (MV), Embolization (embolization), and Vasopressor Use (Vaso). Trauma (trauma), Hypertension (HTN), Diabetes Mellitus (DM), and Craniotomy (craniotomy) were additionally retained a priori for clinical relevance. "Selected" denotes CIE retention, "Selected VIF" denotes retention after VIF screening, and "Collinearity" indicates whether the prespecified VIF threshold was exceeded. Subsequent multivariable models use these abbreviations.

Supplementary Table 9. Sensitivity Analysis Using TyG Trajectories Defined by 10 Sequential Measurements

Variable	Model 1		Model 2		Model 3	
	HR (95% CI)	*P*	HR (95% CI)	*P*	HR (95% CI)	*P*
**Time interval: 0–7 days**						
**Low–slightly increasing**	Reference		Reference		Reference	
**Moderate–increasing**	1.45 (0.94–2.24)	0.094	1.33 (0.85–2.09)	0.218	1.03 (0.64–1.63)	0.917
**Persistently High**	1.11 (0.77–1.58)	0.578	1.03 (0.71–1.49)	0.892	0.89 (0.61–1.30)	0.552
**Time interval: >7 days**						
**Low–slightly increasing**	Reference		Reference		Reference	
**Moderate–increasing**	1.78 (1.38–2.30)	<0.001	1.71 (1.31–2.24)	<0.001	1.42 (1.07–1.87)	0.014
**Persistently High**	1.75 (1.41–2.16)	<0.001	1.77 (1.43–2.21)	<0.001	1.63 (1.30–2.05)	<0.001

Note: Cox models were time-stratified at day 7 (0–7 vs >7 days). TyG trajectories were re-estimated using 10 sequential TyG measurements and contrasted with Low–slightly increasing (LSI) as reference (Moderate–increasing [MI] and Persistently High [PH]). Model 1 unadjusted; Model 2 and Model 3 correspond to the partially and fully adjusted covariate sets specified in Table 2.

Supplementary Table 10. Sensitivity Analyses Using Alternative Time-Stratification Schemes

Variable	Model 1		Model 2		Model 3	
	HR (95% CI)	*P*	HR (95% CI)	*P*	HR (95% CI)	*P*
**Time interval: 0–3 days**						
**Low–slightly increasing**	Reference		Reference		Reference	
**Moderate–increasing**	0.54 (0.22–1.30)	0.169	0.48 (0.21–1.20)	0.119	0.42 (0.17–1.00)	0.050

**Persistently High**	0.72 (0.28–1.9)	0.491	0.65 (0.25–1.68)	0.372	0.52 (0.20–1.35)	0.177
**Time interval: >3 days**						
**Low–slightly increasing**	Reference		Reference		Reference	
**Moderate–increasing**	1.62 (1.33–1.97)	<0.001	1.50 (1.22–1.83)	<0.001	1.37 (1.12–1.68)	0.002
**Persistently High**	1.79 (1.45–2.21)	<0.001	1.68 (1.35–2.11)	<0.001	1.43 (1.14–1.79)	0.002
**Time interval: 0–5 days**						
**Low–slightly increasing**	Reference		Reference		Reference	
**Moderate–increasing**	1.01 (0.62–1.63)	0.968	0.77 (0.47–1.25)	0.747	0.92 (0.57–1.50)	0.284
**Persistently High**	1.40 (0.84–2.32)	0.194	1.00 (0.60–1.68)	0.382	1.26 (0.75–2.10)	0.988
**Time interval: 5–10 days**						
**Low–slightly increasing**	Reference		Reference		Reference	
**Moderate–increasing**	1.25 (0.87–1.82)	0.231	0.97 (0.67–1.41)	0.486	1.14 (0.79–1.66)	0.871
**Persistently High**	1.41 (0.93–2.14)	0.105	1.03 (0.67–1.57)	0.289	1.26 (0.82–1.93)	0.901
**Time interval: 10–14 days**						
**Low–slightly increasing**	Reference		Reference		Reference	
**Moderate–increasing**	1.32 (0.85–2.03)	0.217	1.05 (0.68–1.63)	0.435	1.19 (0.77–1.85)	0.819
**Persistently High**	1.75 (1.11–2.75)	0.016	1.36 (0.86–2.16)	0.048	1.59 (1.00–2.53)	0.193
**Time interval: >14 days**						
**Low–slightly increasing**	Reference		Reference		Reference	
**Moderate–increasing**	2.22 (1.63–3.02)	<0.001	2.13 (1.56–2.91)	<0.001	2.10 (1.54–2.87)	<0.001
**Persistently High**	2.12 (1.51–2.95)	<0.001	1.85 (1.31–2.61)	<0.001	2.09 (1.49–2.94)	<0.001

Note: Cox models were time-stratified using two prespecified schemes: a 3-day cutpoint (0–3 and >3 days) and a multi-interval scheme defined by days 5, 10, and 14 (0–5, 5–10, 10–14, and >14 days). TyG trajectory used Low–slightly increasing (LSI) as the reference; Moderate–increasing (MI) and Persistently High (PH) are shown. Model 1 unadjusted; Model 2 and Model 3 correspond to the partially and fully adjusted covariate sets specified in Table 2.

Supplementary Figure 9. Subgroup Hazard Ratios by TBM8p7

Subgroup	Total	Event (%)	Crude HR (95% CI)	Adjusted HR (95% CI)	*P* for interaction
**Overall**	4760	597 (12.5)	1.79 (1.54~2.09)	1.42 (1.18~1.86)	
**Age**					0.179
**Young**	1829	177 (9.7)	2.31 (1.81~2.96)	1.68 (1.24~2.27)	
**Old**	2931	420 (14.3)	1.64 (1.34~2)	1.29 (1.02~1.64)	
**Trauma**					0.842
**No**	3707	484 (13.1)	1.88 (1.58~2.22)	1.41 (1.15~1.72)	
**Yes**	1053	113 (10.7)	1.51 (1.04~2.17)	1.44 (0.95~2.2)	
**Hypertension**					0.976
**No**	1813	204 (11.3)	1.99 (1.53~2.57)	1.75 (1.24~2.45)	
**Yes**	2947	393 (13.3)	1.68 (1.38~2.03)	1.38 (1.1~1.72)	
**Craniotomy**					0.108
**No**	2812	333 (11.8)	1.78 (1.47~2.15)	1.35 (1.07~1.69)	
**Yes**	1948	264 (13.6)	1.79 (1.36~2.36)	1.69 (1.22~2.33)	
**Cohort**					0.01
**External**	941	223 (23.7)	1.25 (1~1.57)	1.36 (1.06~1.74)	
**Derivation**	3819	374 (9.8)	2.16 (1.66~2.8)	1.52 (1.1~2.09)	

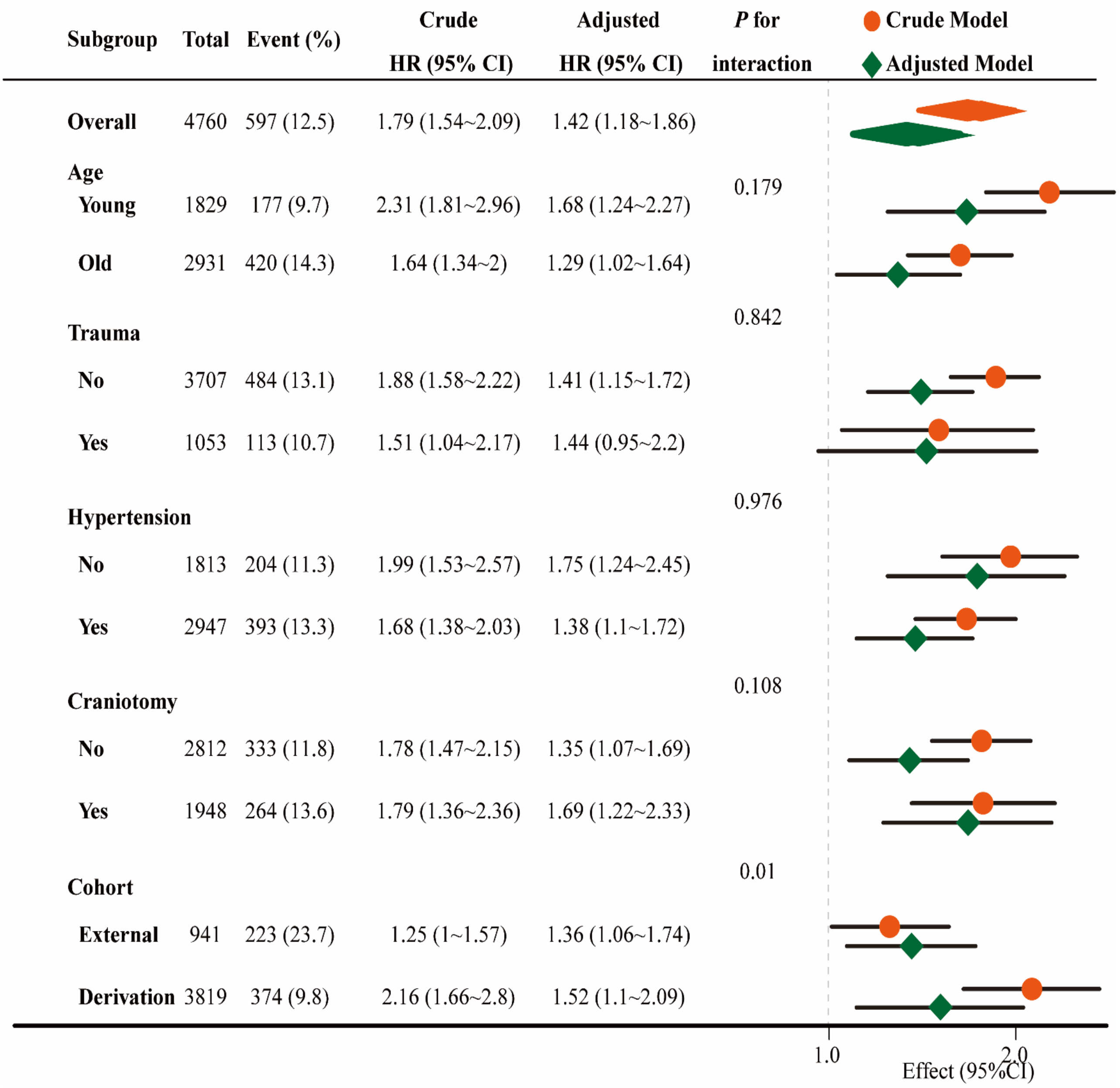

Legend: Forest plots show hazard ratios (HRs) with 95% CIs for TBM8p7 (per 1-unit increase) across prespecified subgroups. Orange circles denote crude HRs; green diamonds denote fully adjusted HRs. Horizontal bars indicate 95% CIs; the vertical dashed line marks HR = 1.

Supplementary Table 11. Baseline Characteristics of the Derivation and External Validation Groups

Characteristics	Total (n = 4760)	External Validation (n = 941)	Derivation (n = 3819)	*P*
**Age (years)**	57.6 ± 14.5	58.9 ± 17.5	57.3 ± 13.7	0.002
**Temp (°C)**	36.6 ± 0.7	36.7 ± 1.0	36.6 ± 0.6	<0.001
**WBC (×10⁹/L)**	11.1 ± 5.0	13.5 ± 7.1	10.5 ± 4.1	<0.001
**Sodium (mmol/L)**	140.7 ± 5.1	140.1 ± 5.9	140.9 ± 4.9	<0.001
**TyG trajectory**				0.116
**Low–slightly increasing**	2940 (61.8)	570 (60.6)	2370 (62.1)	
**Moderate–increasing**	1117 (23.5)	212 (22.5)	905 (23.7)	
**Persistently High**	703 (14.8)	159 (16.9)	544 (14.2)	
**TBM8p7**	0.1 (0.0, 0.5)	0.5 (0.1, 0.9)	0.1 (0.0, 0.3)	<0.001
**INR**	1.1 ± 0.4	1.3 ± 0.7	1.0 ± 0.2	<0.001
**APACHE III**	65.9 ± 19.8	79.1 ± 24.9	62.7 ± 16.8	<0.001
**CCI**	3.0 (2.0, 5.0)	3.0 (1.0, 5.0)	3.0 (2.0, 5.0)	0.001
**GCS**	9.0 ± 5.2	9.2 ± 4.5	9.0 ± 5.4	0.212
**SOFA**	4.0 (2.0, 6.0)	5.0 (2.0, 7.0)	4.0 (1.0, 5.0)	<0.001
**Vaso**	532 (11.2)	231 (24.5)	301 (7.9)	<0.001
**MV**	2353 (49.4)	519 (55.2)	1834 (48.0)	<0.001

Notes: Continuous variables are summarized as mean ± SD or median (IQR); categorical variables as n (%), with binary variables reported as "Yes." Comparisons are between the derivation cohort (NSICU) and the pooled external validation cohort (MIMIC-IV and eICU). TBM8p7 denotes the threshold-based mean metric derived using the prespecified TyG threshold of 8.7. Abbreviations: TyG, triglyceride–glucose index; TBM, threshold-based mean; INR, International Normalized Ratio; APACHE III, Acute Physiology and Chronic Health Evaluation III; CCI, Charlson Comorbidity Index; GCS, Glasgow Coma Scale; SOFA, Sequential Organ Failure Assessment; Vaso, vasopressor; MV, mechanical ventilation; WBC, white blood cell count.

Supplementary Figure 10. Incremental Prediction Gain From Adding TyG Trajectory and TBM8p7

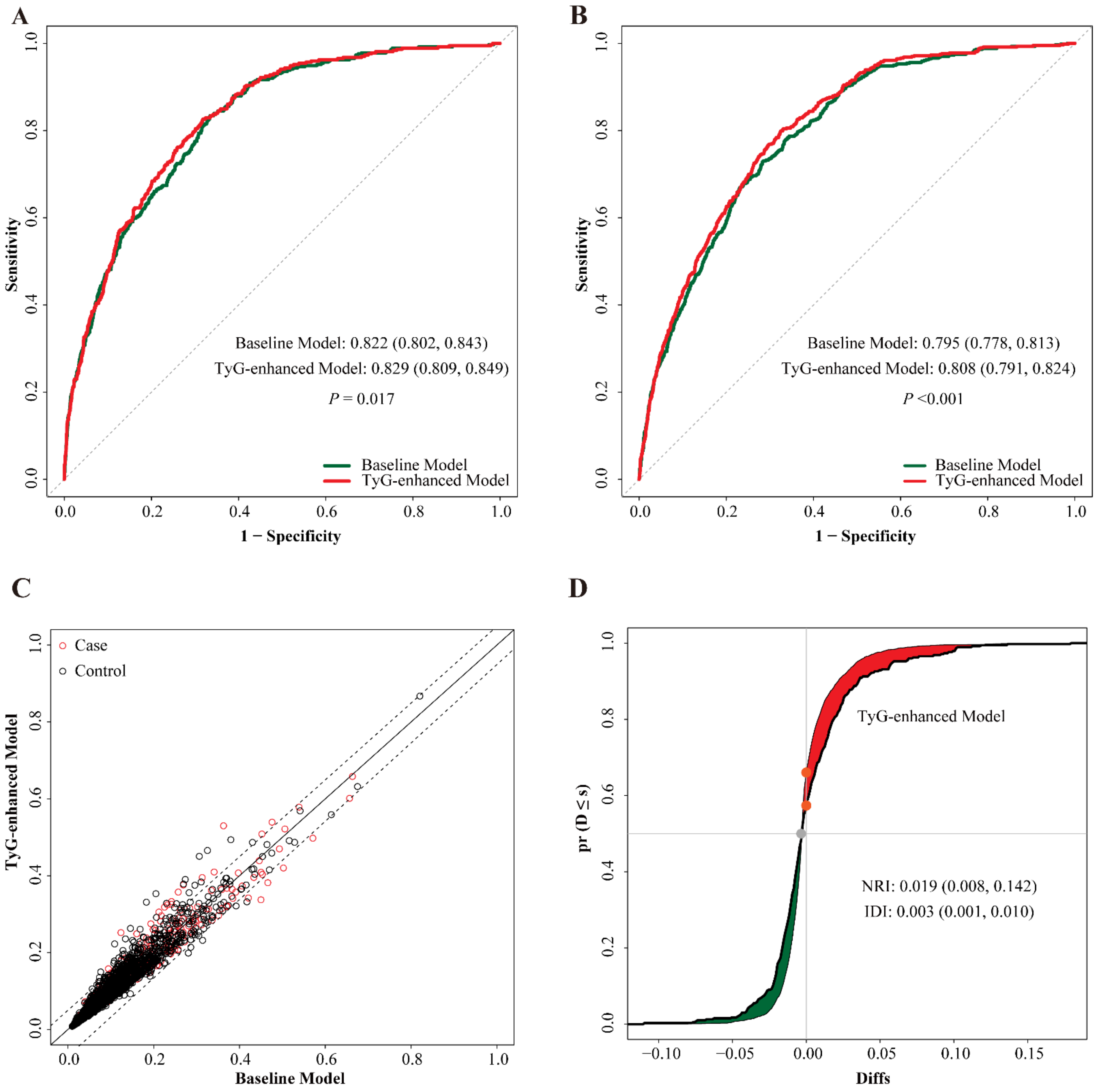

Legend: Panels A–B compare discrimination between a baseline model (Age, temperature, WBC, sodium, INR, APACHE III, CCI, GCS, SOFA, vasopressor use, and MV) and a TyG-enhanced model (baseline + TyG trajectory and TBM8p7) in the integrated cohort (A) and the derivation cohort (B); AUROC (95% CI) and DeLong P values are annotated. Panel C plots individual predicted probabilities from the TyG-enhanced model versus the baseline model (derivation cohort), colored by outcome status. Panel D shows the distribution of individual risk differences (TyG-enhanced − baseline), with NRI and IDI (95% CI) reported.

Supplementary Figure 11. Forest Plot of Cross-Validated Classifier Performance in the Training Set

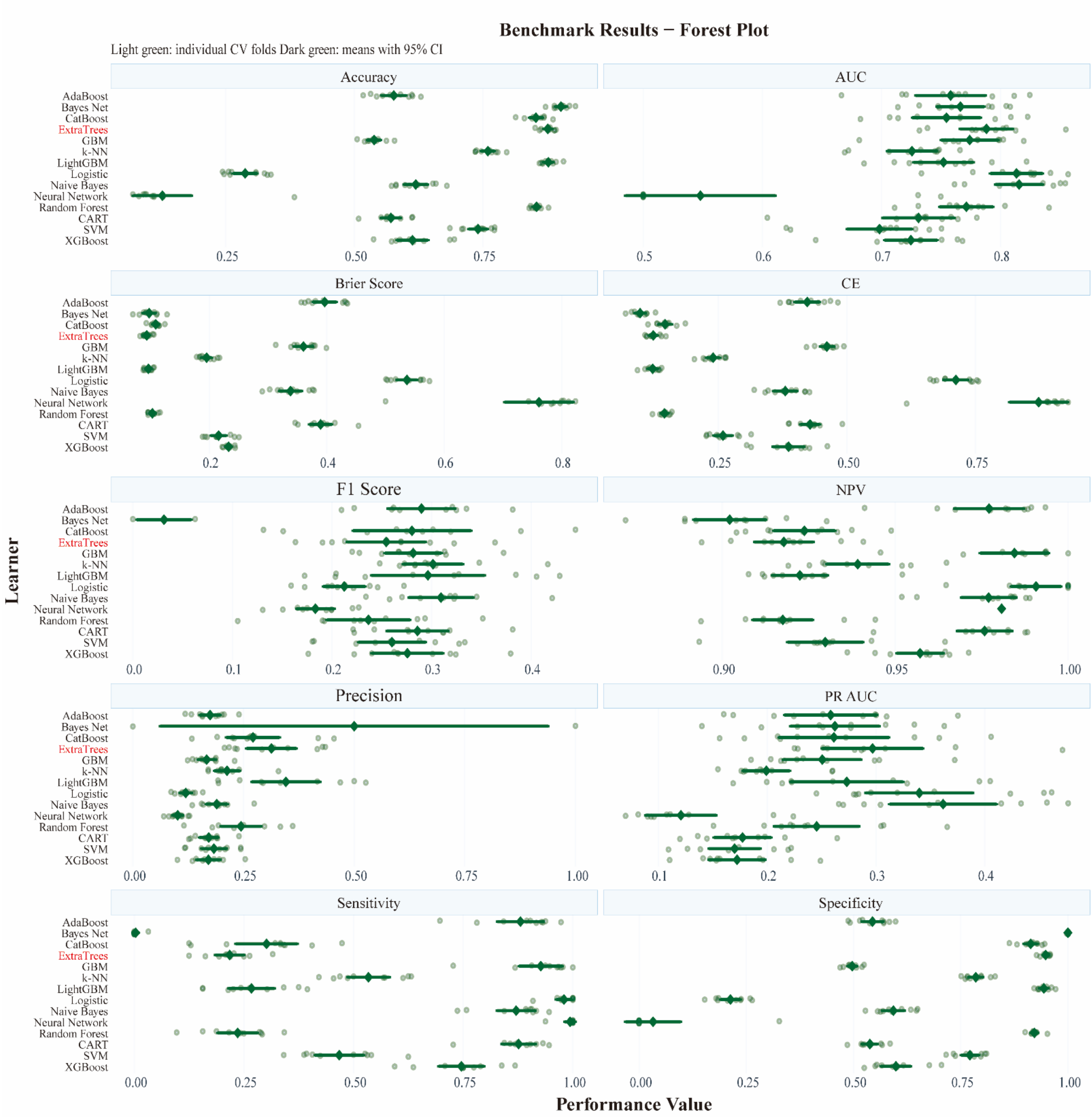

Legend: Stratified 10-fold cross-validation in the training set. For each classifier and metric, light green points show fold-level estimates, and dark green points with horizontal bars show the fold mean and 95% confidence interval in a forest-plot display. Panels report Accuracy, AUC, Brier score, classification error (CE), F1 score, NPV, Precision, PR AUC, Sensitivity, and Specificity. Lower values indicate better performance for Brier score and CE; higher values are better for the remaining metrics.

Supplementary Table 12. Cross-Validated Performance of Benchmark Classifiers in the Training Set

Classifier	AUROC	Accuracy	Brier score	F1 score	CE	PR AUC	Sensitivity	Specificity	NPV	PPV
**AdaBoost**	0.76	0.58	0.40	0.29	0.42	0.26	0.88	0.54	0.98	0.17
**Bayesian Network**	0.77	0.90	0.10	NA	0.10	0.26	0.00	1.00	0.90	NA
**CatBoost**	0.75	0.85	0.11	0.28	0.15	0.26	0.30	0.91	0.92	0.27
**ExtraTrees**	0.79	0.88	0.09	0.25	0.12	0.30	0.22	0.95	0.92	0.31
**GBM**	0.77	0.54	0.36	0.28	0.46	0.25	0.93	0.50	0.98	0.17
**k-NN**	0.73	0.76	0.20	0.30	0.24	0.20	0.53	0.79	0.94	0.21
**LightGBM**	0.75	0.88	0.10	0.30	0.12	0.27	0.27	0.94	0.92	0.35
**Logistic**	0.81	0.29	0.54	0.21	0.71	0.34	0.98	0.21	0.99	0.12
**Naive Bayes**	0.82	0.62	0.34	0.31	0.38	0.36	0.87	0.59	0.98	0.19
**Neural Network**	0.55	0.13	0.76	0.18	0.87	0.12	0.99	0.03	NA	0.10
**Random Forest**	0.77	0.85	0.10	0.24	0.15	0.25	0.24	0.92	0.92	0.24
**CART**	0.73	0.57	0.39	0.29	0.43	0.18	0.88	0.54	0.98	0.17
**SVM**	0.70	0.74	0.22	0.26	0.26	0.17	0.47	0.77	0.93	0.18
**XGBoost**	0.72	0.61	0.23	0.28	0.39	0.17	0.75	0.60	0.96	0.17

Note: Values are means across stratified 10-fold cross-validation in the training set. AUROC and PR AUC assess discrimination; the Brier score reflects probabilistic error (lower is better); CE is classification error (CE = 1 − Accuracy); F1 is the harmonic mean of PPV (Precision) and Sensitivity (Recall). Higher is better for AUROC, PR AUC, F1, Sensitivity, Specificity, PPV, and NPV; lower is better for CE and Brier score. NA indicates folds where a classifier predicted a single class, rendering PPV/NPV or F1 undefined. Abbreviations: CART, Classification and Regression Tree; GBM, Gradient Boosting Machine; k-NN, k-Nearest Neighbors; SVM, Support Vector Machine; PPV, positive predictive value; NPV, negative predictive value.

Supplementary Table 13. Performance of Benchmark Classifiers on the Internal Test Set

Classifier	AUROC	Accuracy	Brier score	F1 score	CE	PR AUC	Sensitivity	Specificity	NPV	PPV
**AdaBoost**	0.82	0.54	0.41	0.29	0.46	0.30	0.95	0.50	0.99	0.17
**Bayesian Network**	0.76	0.90	0.10	NA	0.10	0.29	0.00	1.00	0.90	NA
**CatBoost**	0.82	0.87	0.09	0.37	0.13	0.36	0.38	0.93	0.93	0.36
**ExtraTrees**	0.83	0.87	0.09	0.29	0.13	0.36	0.27	0.94	0.92	0.31
**GBM**	0.78	0.51	0.38	0.26	0.49	0.27	0.92	0.46	0.98	0.15
**k-NN**	0.76	0.77	0.20	0.32	0.23	0.21	0.54	0.80	0.94	0.22
**LightGBM**	0.83	0.88	0.09	0.41	0.12	0.41	0.43	0.93	0.94	0.39
**Logistic**	0.87	0.30	0.54	0.22	0.70	0.37	1.00	0.22	1.00	0.12
**Naive Bayes**	0.88	0.70	0.25	0.37	0.30	0.41	0.92	0.68	0.99	0.23
**Neural Network**	0.50	0.10	0.80	0.18	0.90	0.10	1.00	0.00	NA	0.10
**Random Forest**	0.82	0.85	0.10	0.24	0.15	0.27	0.24	0.92	0.92	0.24
**CART**	0.74	0.54	0.40	0.28	0.46	0.18	0.92	0.50	0.98	0.17
**SVM**	0.70	0.71	0.23	0.27	0.29	0.22	0.54	0.73	0.94	0.18
**XGBoost**	0.68	0.54	0.24	0.24	0.46	0.14	0.76	0.52	0.95	0.14

Note: Performance metrics were evaluated on the held-out internal test set using the final classifiers fitted on the entire training set.

Supplementary Table 14. Performance of Benchmark Classifiers on the External Validation Set

Classifier	AUROC	Accuracy	Brier score	F1 score	CE	PR AUC	Sensitivity	Specificity	NPV	PPV
**AdaBoost**	0.61	0.50	0.43	0.42	0.50	0.31	0.76	0.42	0.85	0.29
**Bayesian Network**	0.62	0.75	0.24	0.29	0.26	0.37	0.22	0.91	0.79	0.43
**CatBoost**	0.63	0.73	0.21	0.29	0.27	0.32	0.23	0.88	0.79	0.38
**ExtraTrees**	0.67	0.74	0.18	0.18	0.26	0.35	0.12	0.93	0.77	0.34
**GBM**	0.63	0.44	0.43	0.42	0.56	0.33	0.85	0.31	0.87	0.28
**k-NN**	0.63	0.61	0.33	0.42	0.39	0.31	0.59	0.61	0.83	0.32
**LightGBM**	0.64	0.74	0.20	0.34	0.26	0.37	0.28	0.88	0.80	0.43
**Logistic**	0.66	0.29	0.58	0.40	0.71	0.34	0.99	0.08	0.95	0.25
**Naive Bayes**	0.71	0.28	0.59	0.39	0.72	0.43	0.97	0.07	0.89	0.24
**Neural Network**	0.50	0.24	0.67	0.38	0.76	0.24	1.00	0.00	NA	0.24
**Random Forest**	0.67	0.76	0.17	0.14	0.24	0.35	0.09	0.97	0.77	0.44
**CART**	0.60	0.45	0.48	0.41	0.55	0.29	0.79	0.35	0.84	0.27
**SVM**	0.53	0.66	0.30	0.23	0.34	0.25	0.22	0.79	0.77	0.25
**XGBoost**	0.58	0.52	0.25	0.37	0.48	0.29	0.60	0.49	0.80	0.27

Note: Performance metrics were evaluated on the independent external validation cohort using the same final classifiers, without model refitting or threshold recalibration.

Supplementary Table 15. Final Hyperparameters of the Tuned ExtraTrees Classifier

Parameter	Value
**classif.extratrees.ntree**	595
**classif.extratrees.mtry**	1
**classif.extratrees.nodesize**	5
**classif.extratrees.numRandomCuts**	1
**classif.extratrees.evenCuts**	FALSE
**classif.extratrees.numThreads**	1
**classif.extratrees.numRandomTaskCuts**	1

Note: Hyperparameters correspond to the locked final ExtraTrees model after tuning within the NSICU training set using stratified 10-fold cross-validation.

Supplementary Table 16. Sensitivity Analysis of the Tuned ExtraTrees Classifier After Youden Index–Based Threshold Optimization

Cohort	AUROC	Accuracy	Brier score	F1 score	CE	PR AUC	Sensitivity	Specificity	NPV	PPV
**CV**	0.79	0.66	0.09	0.31	0.34	0.28	0.78	0.64	0.96	0.19
**Training**	1.00	1.00	0.00	1.00	0.00	1.00	1.00	1.00	1.00	1.00
**Internal test**	0.83	0.68	0.09	0.36	0.32	0.35	0.92	0.66	0.99	0.22
**External validation**	0.66	0.48	0.18	0.44	0.52	0.34	0.86	0.36	0.89	0.29

Notes: Metrics are reported for the final ExtraTrees classifier after hyperparameter tuning on the NSICU training set. “CV” values are out-of-fold means from stratified 10-fold cross-validation and reflect expected performance on unseen data. The “Training” row reports apparent (in-sample) performance of the fitted model. Internal test and external validation metrics were obtained out-of-sample using the same fitted model and a Youden index–optimized operating threshold of 0.118 derived from cross-validation.

Supplementary Figure 12. Internal Test Set Performance of the ExtraTrees Classifier

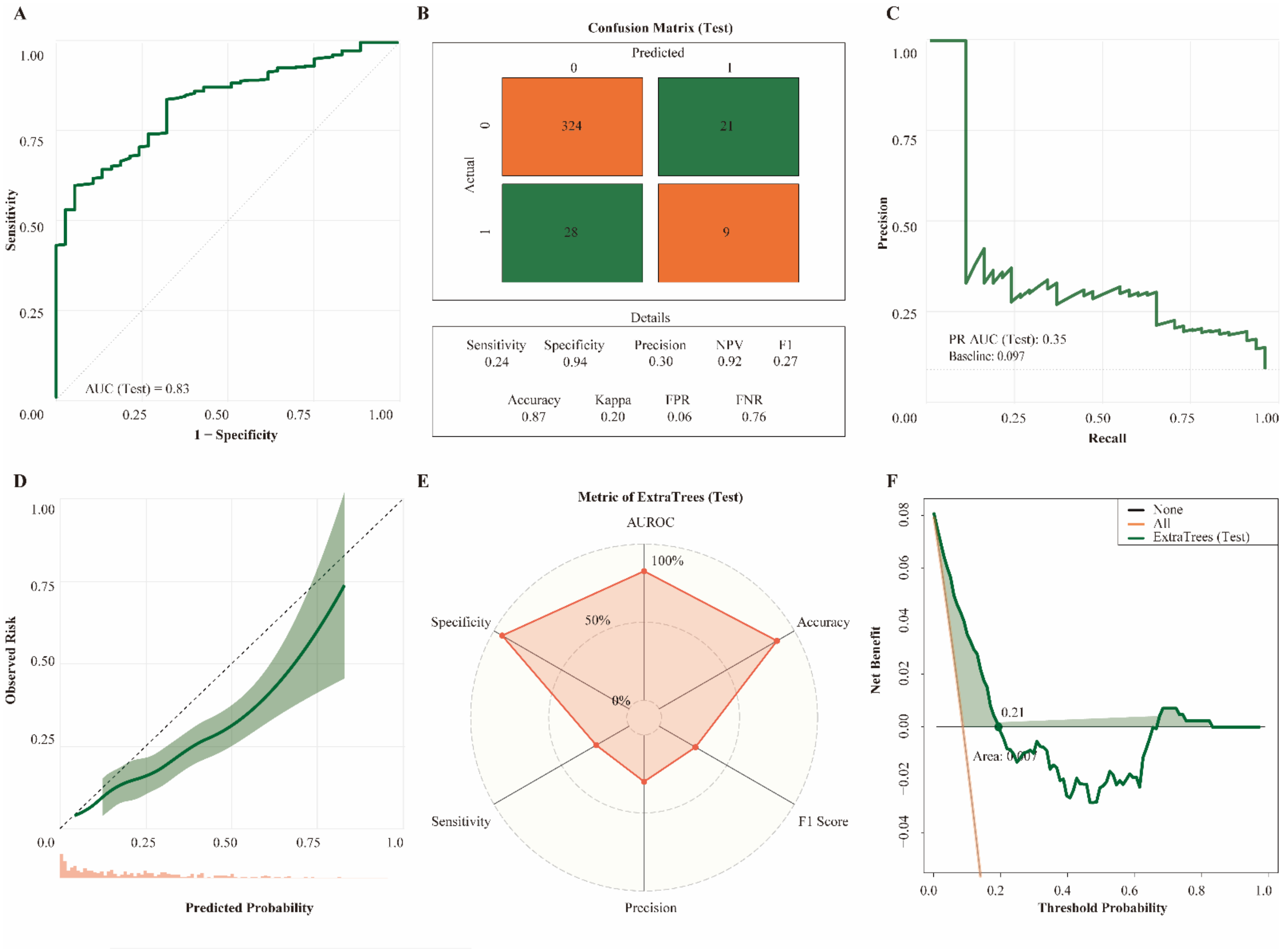

Legend: Panels A–F report the ExtraTrees model evaluated on the internal test set (hold-out split from the derivation cohort). Solid lines are point estimates; shaded ribbons indicate 95% CIs. A, ROC curve (test set AUC). B, Confusion matrix with Accuracy, Sensitivity (Recall), Specificity, Precision (PPV), F1 score, and Cohen's κ (chance-corrected agreement). C, Precision–recall curve (test set PR AUC); the horizontal dashed line marks the outcome prevalence. D, Calibration: LOESS-smoothed observed vs predicted risk with the 45° reference line; histogram of predicted probabilities shown below. E, Metric profile (radar) summarizing AUC, Accuracy, Sensitivity, Specificity, Precision, and F1. F, Decision-curve analysis: net benefit vs Threshold probability with "Treat none" and "Treat all" reference strategies.

Supplementary Figure 13. External Validation Performance of the ExtraTrees Classifier

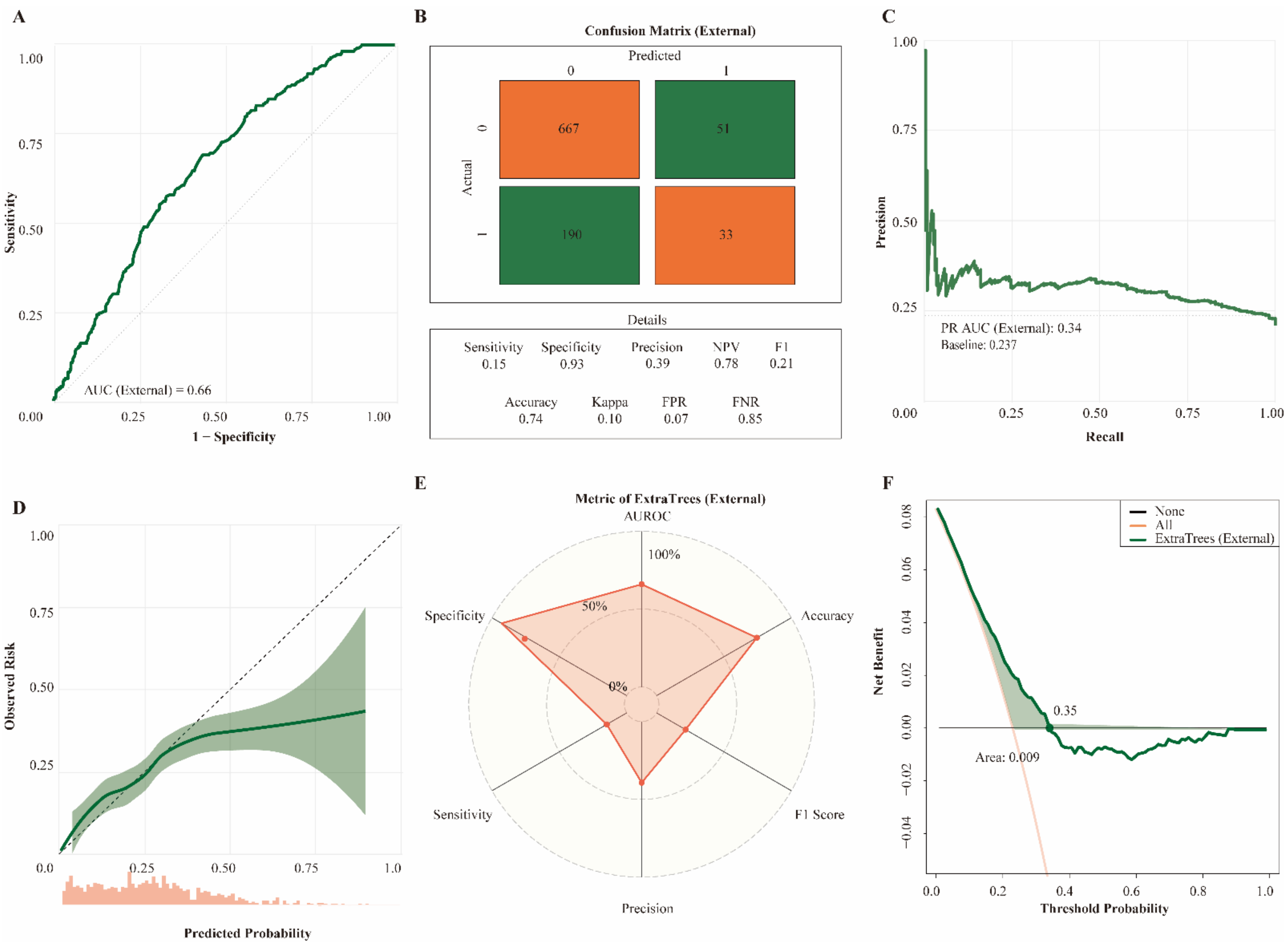

Legend: Panels A–F (ROC, confusion matrix, PR curve, calibration, radar, DCA) summarize ExtraTrees on the external validation cohort; solid lines = point estimates, ribbons = 95% CIs; PR dashed line = prevalence; DCA x-axis = threshold probability

Supplementary Figure 14. Loss-Based Feature Importance for the ExtraTrees Classifier in the Training and Internal Test Sets

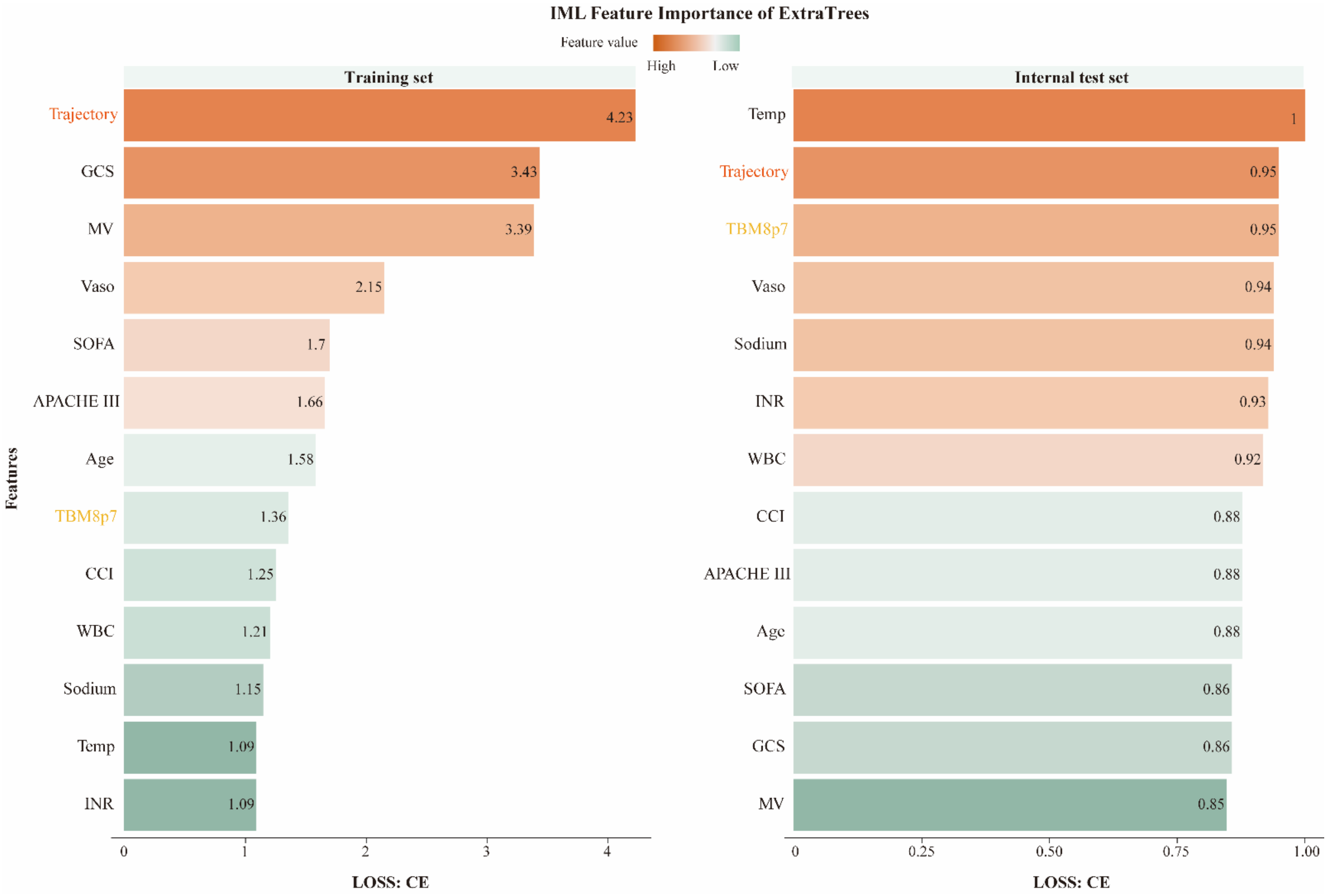

Legend: Variable importance was computed with an interpretable machine-learning (IML) approach that measures the increase in classification error (CE) after perturbing each feature (larger CE increase = higher importance). The left panel shows the model fitted on the training set; the right panel shows the same procedure applied to the internal test set. Dynamic metabolic features (TyG trajectory and TBM8p7) remained within the upper importance tier together with neurologic status (GCS), organ support (MV, vasopressors), and global severity scores (SOFA, APACHE III).

Supplementary Figure 15. Permutation Importance and Case-Level Explanations for the ExtraTrees Classifier

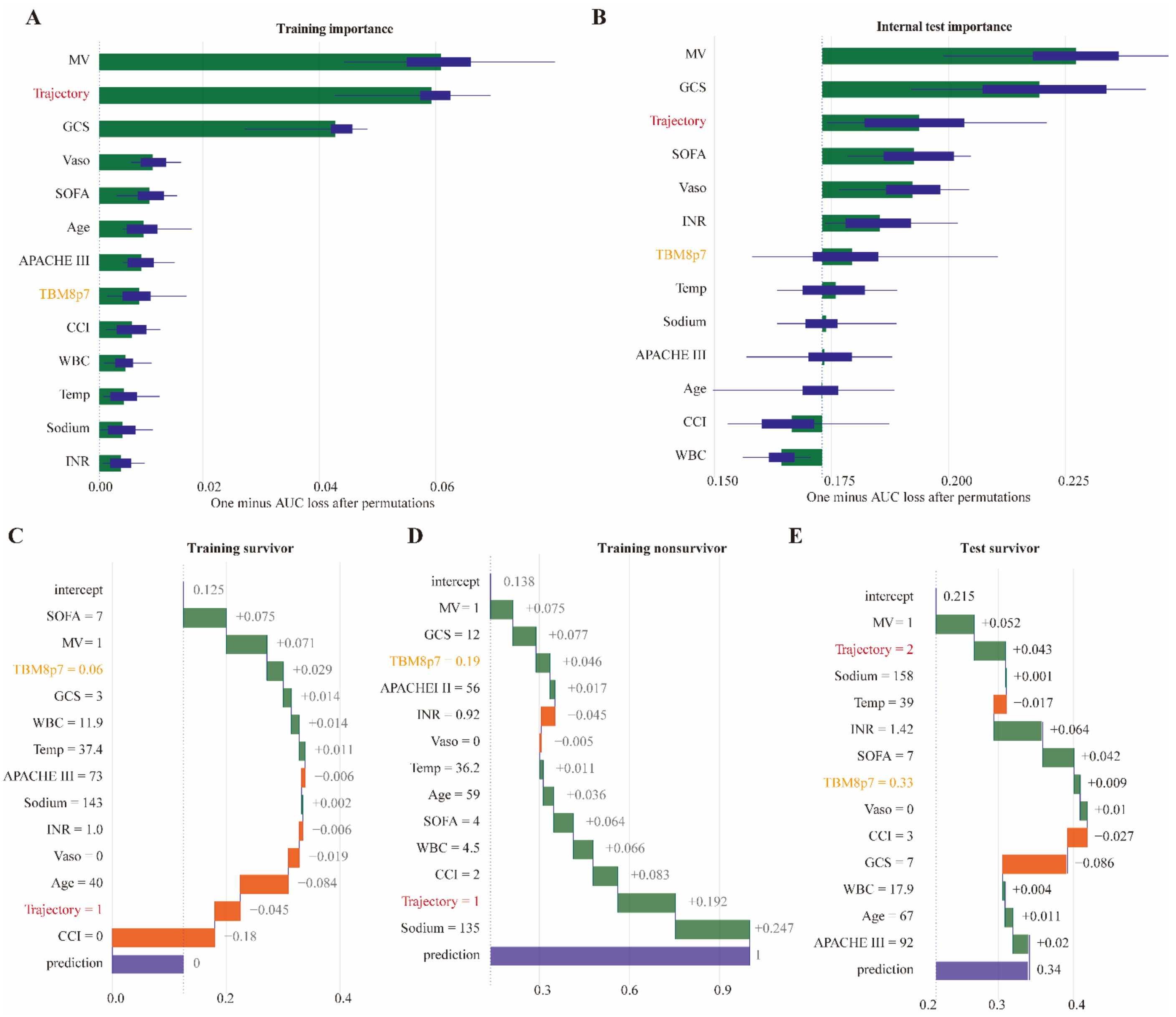

Legend: (A–B) Permutation importance was calculated as the mean decrease in AUROC after 100 random shuffles of each variable, evaluated on the training set (A) and on the internal test set (B). TyG trajectory and TBM8p7 are highlighted as the two dynamic metabolic features introduced in this study. (C–E) Example breakdown profiles decompose individual predictions into additive contributions relative to the model baseline: a training survivor (C), a training non-survivor (D), and an internal-test survivor (E).

Supplementary Figure 16. Web-Based Prediction Tool for Individualized Risk and Explanation

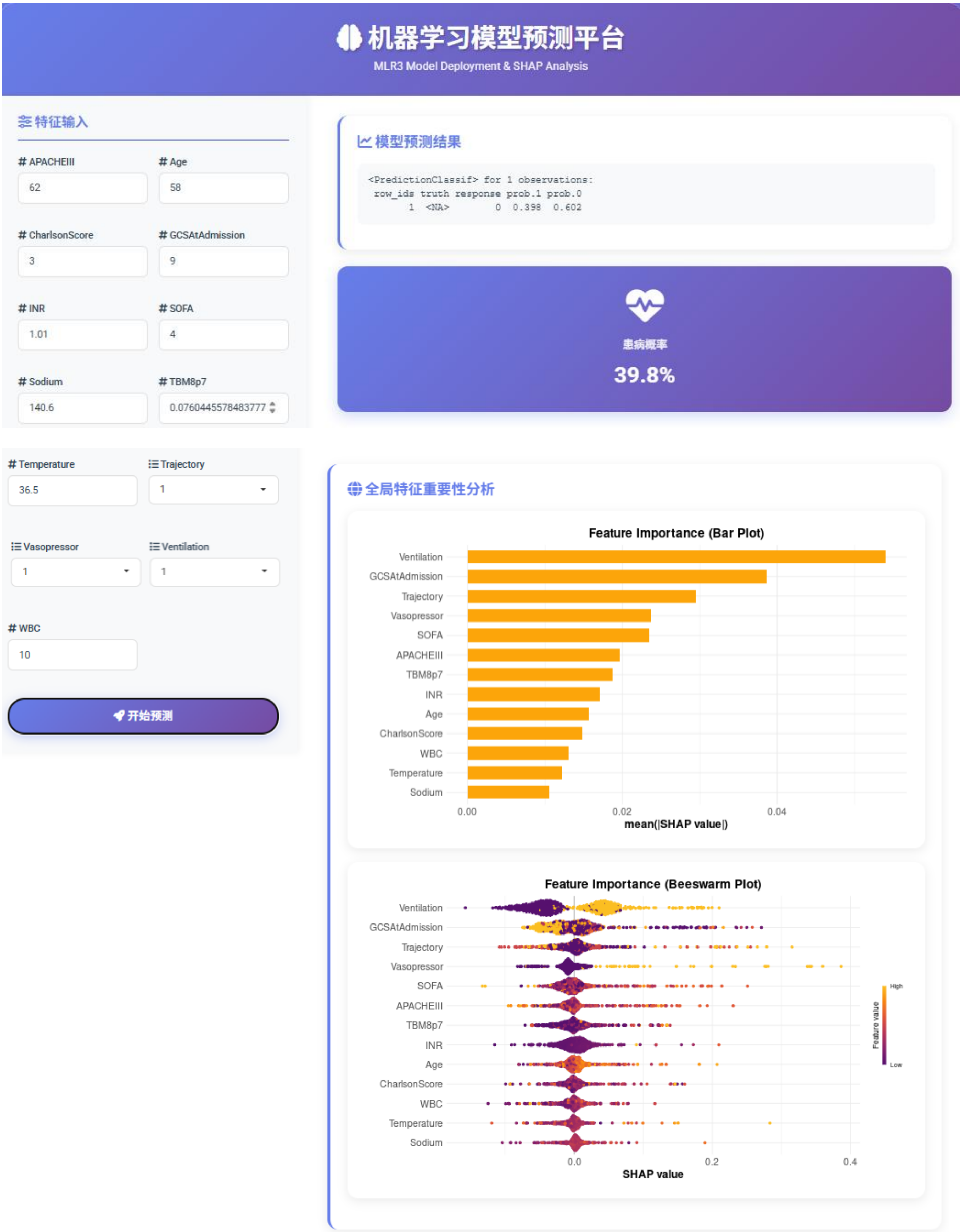

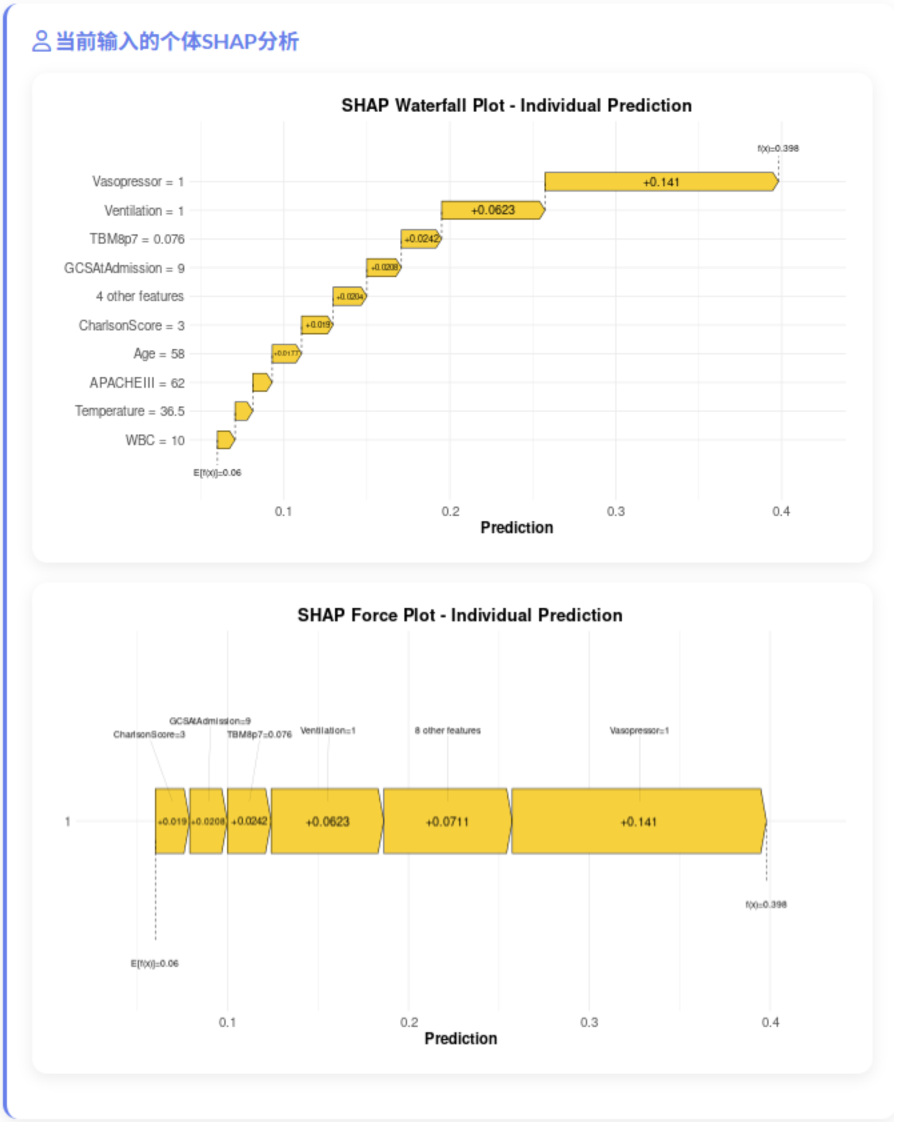

Legend: Screenshot of the public calculator (URL：https://njudrumtowernsicu.shinyapps.io/ABI_Prognosis_ModelTyG/) implementing the finalized ExtraTrees classifier. Users enter baseline variables on the left. The app returns the predicted in-hospital mortality risk (top right) and case-level SHAP explanations: a waterfall plot showing each feature’s contribution from the baseline risk to the final prediction (positive = yellow bars, negative = purple bars) and a force plot summarizing the same directionality. The deployed model corresponds to that developed in the derivation cohort and validated on internal and external cohorts; the app does not retain identifiable inputs on the server. The calculator is provided for research/educational use and should not be used as a sole basis for clinical decisions.
